# Active and secreted IgA-coated bacterial fractions from the human gut reveal an under-represented microbiota core

**DOI:** 10.1038/srep03515

**Published:** 2013-12-17

**Authors:** Giuseppe D'Auria, Francesc Peris-Bondia, Mária Džunková, Alex Mira, Maria Carmen Collado, Amparo Latorre, Andrés Moya

**Affiliations:** 1 Joint Unit of Research in Genomics and Health, Centre for Public Health Research (CSISP) - Cavanilles Institute for Biodiversity and Evolutionary Biology (University of Valencia), Valencia, 46020; Spain; 2 CIBER Epidemiología y Salud Pública (CIBERESP); Spain; 3 The Institute of Agrochemistry and Food Technology, Spanish National Research Council (IATA-CSIC), Valencia, 46100; Spain; 4 These authors contributed equally to this work.

## Abstract

Host-associated microbiota varies in distribution depending on the body area inhabited. Gut microbes are known to interact with the human immune system, maintaining gut homoeostasis. Thus, we studied whether secreted-IgA (S-IgA) coat specific microbial taxa without inducing strong immune responses. To do so, we fractionated gut microbiota by flow cytometry. We found that active and S-IgA-coated bacterial fractions were characterized by a higher diversity than those observed in raw faecal suspensions. A long-tail effect was observed in family distribution, revealing that rare bacteria represent up to 20% of total diversity. While *Firmicutes* was the most abundant phylum, the majority of its sequences were not assigned at the genus level. Finally, the single-cell-based approach enabled us to focus on active and S-IgA-coated bacteria. Thus, we revealed a microbiota core common to the healthy volunteers participating in the study. Interestingly, this core was composed mainly of low frequency taxa (e.g. *Sphingomonadaceae*).

The human gastrointestinal tract (GIT) hosts a complex bacterial community which, for decades, has been subject to extensive study involving both culture-dependent and -independent methods^1^,^2^,^3^,^4^. The GIT microbiota is acquired very early in a month-scale laps from the time of delivery of the baby^5^. Together with this very short time scale, the “inoculum” is determined by the mode of delivery (vaginal or C-section), by the mode of feeding (breast milk or formula) and by environmental factors at the time of delivery, among others^6^,^7^,^8^.

Most likely, very few organisms of maternal origin will be maintained in the newborn after the first month of life^9^,^10^; after this short time, the intestine already hosts its own microbiota, which undergoes complex bacterial turnover until reaching a stable composition that is then fairly constant for life^11^. To achieve this homoeostasis, innate and adaptive immune responses work together. Growing evidence shows a parallel evolution of the immune system with members of gastrointestinal microbiota^12^,^13^,^14^. Thus, since the first day of life, Toll-like receptors (TLR1 to TLR9) are adapted and can recognize specific microbial structures such as lipopolysaccharide (LPS)^15^. Lotz and collaborators described how new-born mice go through a process of tolerance acquisition to LPS expressing bacteria within one hour from delivery; this process contributes to establishing microbiota-host homoeostasis early after delivery^16^. In a very well-designed study, Giraud and collaborators demonstrated that *Escherichia coli* MG1655 inoculated in 8 to12-week old gnotobiotic mice evolves rapidly throughout a fitness process based on mutations and adaptation to the new environment, altering the EnvZ/OmpR operon in 90% of bacteria harvested from independent mice faeces after wild-type “inoculum”^17^. On the other hand, the immune system has to adapt to microbial invasion, avoiding complete annihilation of the intruders, which have to be recognized as the second part of the mutualistic relationship. These interactions are maintained throughout life. In this scenario, the immune system is continuously stimulated, although controlled, avoiding abnormal responses to commensal or potential pathogens which pass through the GIT. For instance, some bacteria such as *Sphingomonas* members have been identified as players in the maintenance of immune response producing antigenic glycosphingolipids which have been correlated with the expansion of invariant natural killer T cells^18^.

Already in 1987, Conley & Delacroix estimated that in the human intestine approximately 40 mg/kg (body weight) are produced every day^19^. It was estimated that in healthy conditions about 36% of GIT microbiota is covered by IgA and that this number can increase up to 69% during inflammatory processes^20^.

A hot research topic involves understanding the role of dominant bacteria and their relationships with rare ones. In a previous work, we highlighted that low frequency taxa are present and are made mainly by active bacteria whose diversity is statistically hidden by the dominant majority^21^. The gastrointestinal tract is the richest body area in terms of microbial groups. The most represented taxa are also the most commonly shared between different samples. However, most of the yet-unknown taxa belong to the under-represented fraction of the gut microbiota^22^,^23^.

While next-generation DNA sequencing methodologies shed light on the enormous variety of bacteria inhabiting the human body, the direct cell-by-cell understanding of which the active players are and which of them interact with the immune system still represents a research challenge. Previously, Van Der Waaij and collaborators (1994) described a method for human faecal microbiota sorting based on fluorescent labelling of anti human IgA^24^. We used an interdisciplinary single-cell approach involving RNA cell staining as a measure of activity^25^,^26^, anti human IgA fluorescent hybridisation, flow cytometry and cell sorting, 16S rDNA amplification and deep pyrosequencing to shed light on different segments of gut microbiota, such as the potential activity of under-represented bacteria. We observed that a long tail of low frequency taxa (below 1% in richness) could involve up to 20% of the active population. We also analysed active and S-IgA-coated bacteria from healthy volunteers which had not received antibiotic treatment and were apparently healthy. Our aim was to determine whether IgA were coating specific taxa. We identified several groups, such as members of *Actinobacteria* or *Sphingobacteria* for which there are already hypotheses regarding their role and activity, confirmed in murine models as active players in gut microbiota and/or as part of the S-IgA-coated fraction.

## Results

### Sequencing and diversity overview

From each sample and fraction we obtained an average of 6,980 sequences over 200 bp (min: 727; max: 14,359 reads). The average read length was 422 bp (+/−89 bp). The number of reads per sample/fraction, as well as the number of particles (cells) sorted by flow cytometry and the ratio with respect to the total number are reported in Table 1. As can be observed, the fraction of active cells ranged from 34% (sample V2) to 66% (V4) with respect to the total community and the proportion of S-IgA-coated bacteria ranged from 21% (V4) to 98% (V3).

Diversity analysis at the genus level showed that FS fractions present a lower evenness with respect to almost all Act samples (V2, V3, V4, V5 and V6) and all IgA fractions, as revealed by the means of the Shannon index. The richness estimator Chao1 detected different behaviours of FS with respect to Act and IgA, being lower in samples V1, V2, V3, V5 and V6 and higher in V4 (see Table 1).

**Figure 1:**
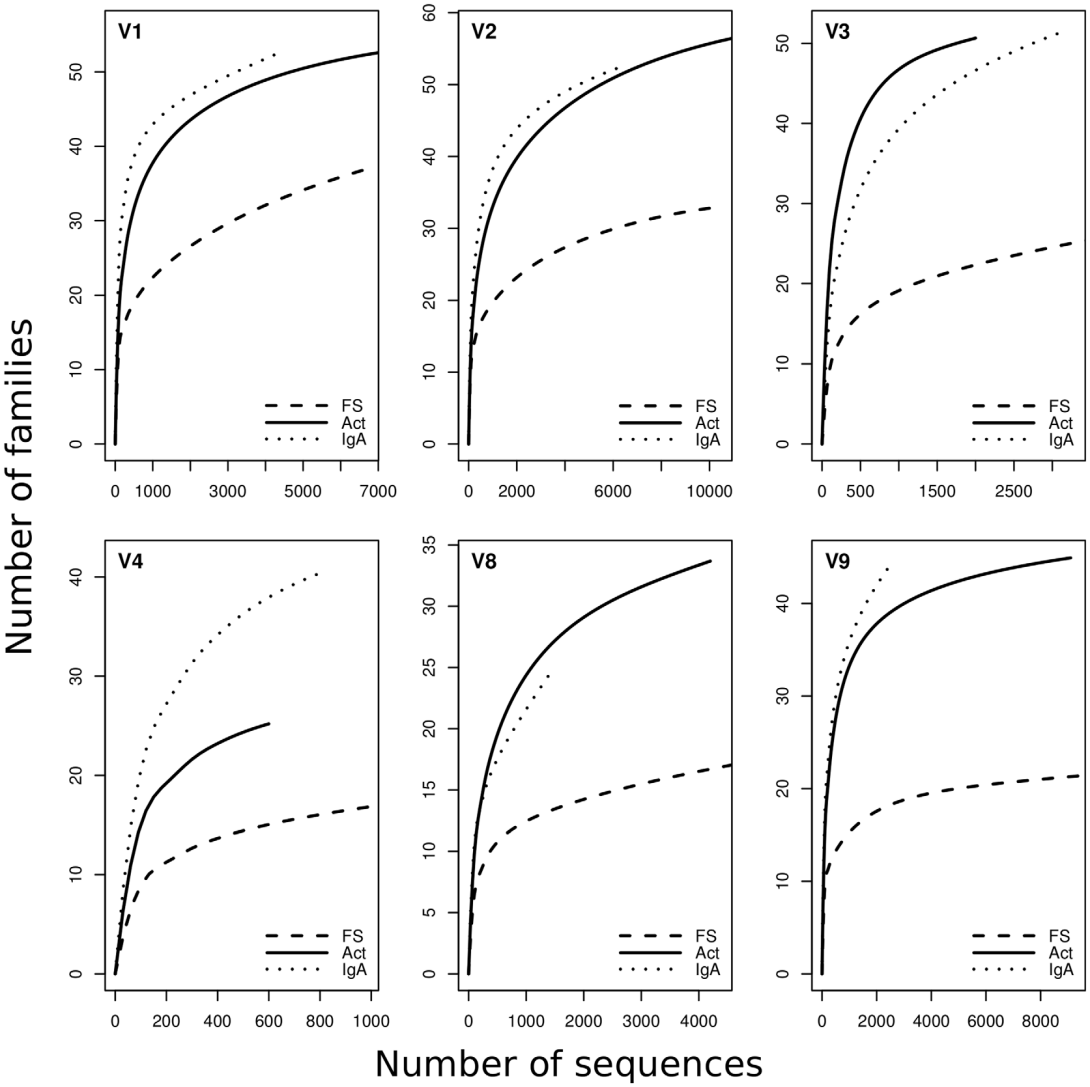
Rarefaction curves calculated for each sample/fraction. FS: faecal suspension. Act: active fractions. IgA: S-IgA-coated bacterial fractions. Every plot corresponds to a volunteer. X axis represents the number of sequences while Y axis represents the number of families encountered.

**Figure 2:**
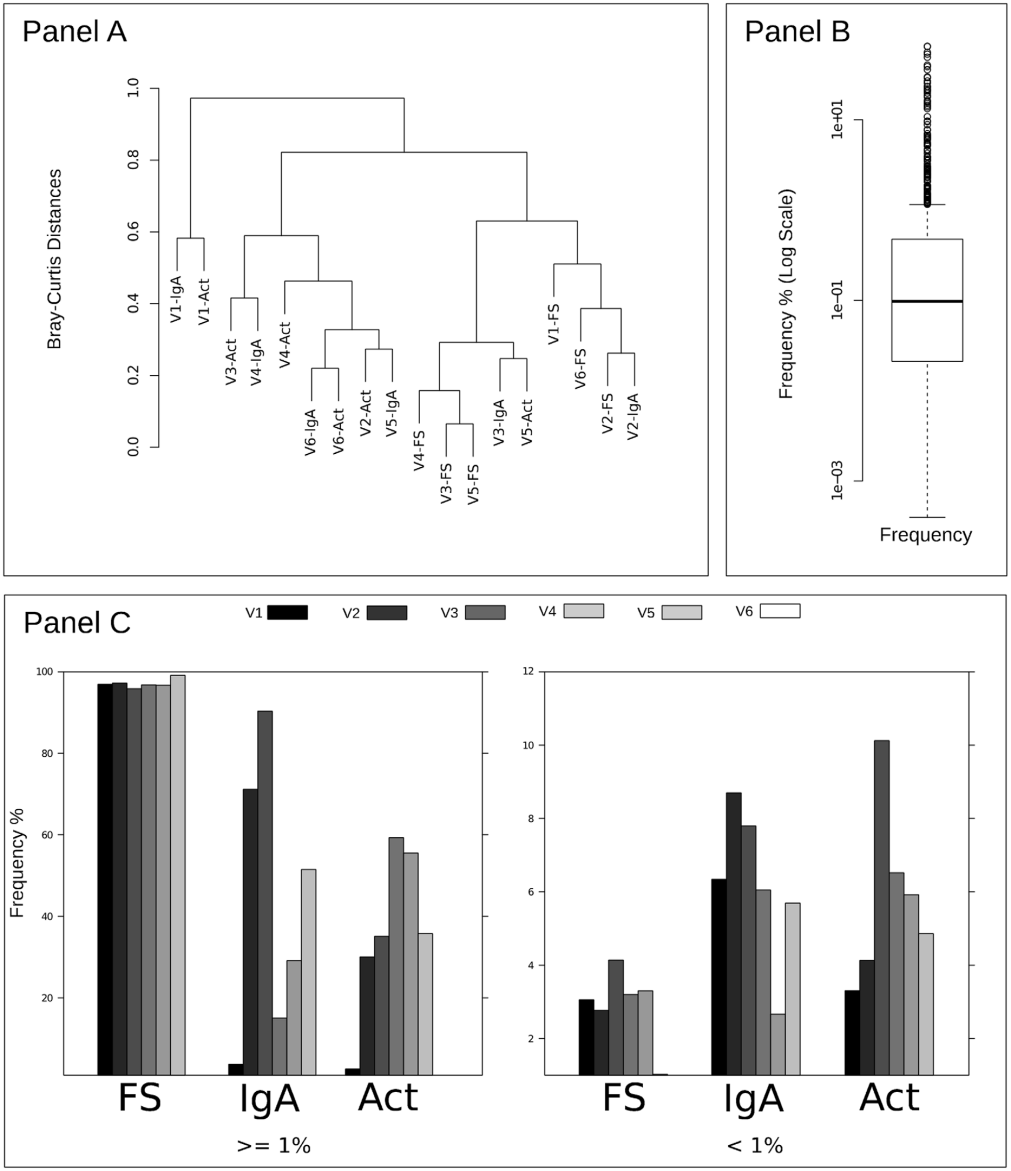
Frequency distributions at family taxonomic rank level. Panel (A) shows clustering of samples/fractions at family level using Bray-Curtis distance matrix and complete linkage clustering method. Panel (B) shows the distribution of all frequency values calculated at family level, Y axis is represented in logarithmic scale. Panel (C) shows aggregation of dominant and rare families, whose abundance values are equal or higher than 1% or lower than 1% respectively. Bars define samples while bar groups define fractions (see legend above the panel).

**Figure 3:**
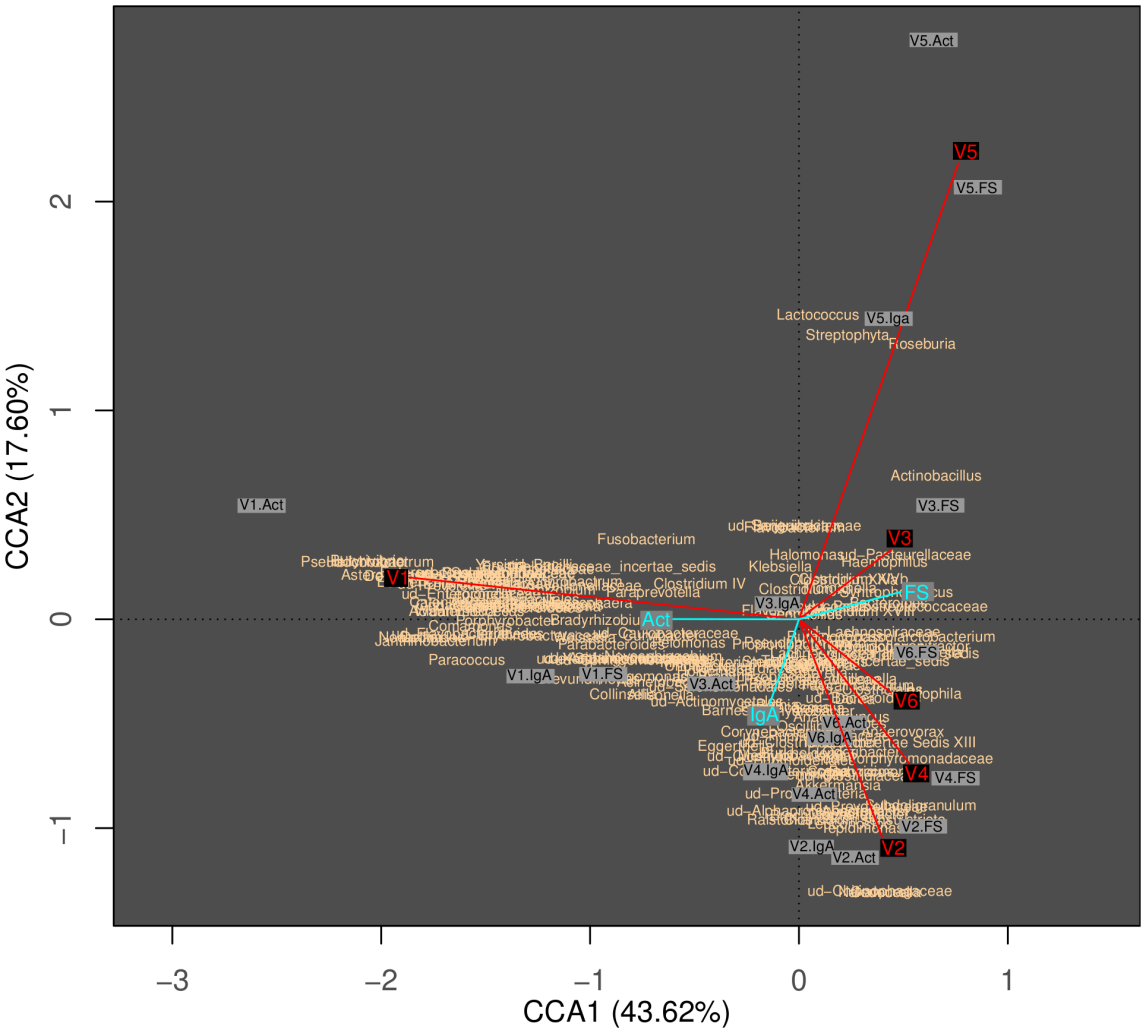
Canonical correspondence analysis. The distribution has been obtained from the Bray Curtis distance matrix, calculated with the genus contingency table. Axes represent distance values on coordinates 1 and 2. Red lines and labels separate samples, light blue lines separates fractions. Gray labels with “Ssample.Fraction” indications describe the distribution of each experiment. Pink labels represent the distribution of genera.

Rarefaction curves flatten at the family level, and all FS fractions flatten earlier than Act and IgA fractions (see Figure 1). Analysis of family distribution revealed that Act and IgA fractions tend to cluster together (Figure 2, panel A). Moreover, Act or S-IgA-coated bacterial fractions are not representative of the whole community; neither could they be considered a subset of it because of the great richness and evenness of species found. Sample V1 showed a different microbial distribution with respect to the other samples, as also shown in the cluster analysis where V1 fractions behave as an outgroup with respect to the other samples/fractions (Figure 2, panel A). At the level of genera, canonical correspondence analysis among all samples collected showed that two major components yield 43.62% and 17.60% of the observed diversity (Figure 3). As expected, sample V1 is the most divergent and also noteworthy is the clear separation between the three fractions. These results are statistically corroborated by the analysis of variance, using distance matrix by “Adonis” (Permutational Multivariate Analysis of Variance Using Distance Matrices) known also as “Permutational Manova”^28^,^27^ and applying Bray-Curtis distance calculation at the genus taxonomic rank. The analysis showed significant p-values for both fractions and sample grouping criteria (Figure 3 and Supplemental Information, Table 1).

To classify low- and high- frequency families, we applied a method based on average inflection point, as previously described^21^. The frequency distribution per families considering the whole dataset had mean and median values of 1.60% and 0.1%, respectively (see Figure 2 panel B), confirming the numeric importance of rare families. Overall mean values per fraction showed that dominant families represented 97.11% (standard deviation: 1.1), 36.37% (sd: 20.39) and 43.46% (sd: 33.54) of families in FS, Act and IgA fractions, respectively, while rare families (frequency below 1%) among all samples represented on average 2.89% (s.d.: 1.1), 5.81% (s.d.: 2.41) and 6.21% (s.d.: 2.08) of families in FS, Act and IgA fractions, respectively (see Figure 2, panel C). Thus, the low-frequency taxa were extraordinarily diverse and diversity is characterized, as expected, by the “long-tail” effect.

### OTUs^97^ distribution and core microbiota

Using an analogy with the pan-genome concept^29^,^30^ we can define “pan-microbiota” by identifying sequence clusters shared among samples (a “core” set of bacteria) as well as clusters specific to each sample.

**Figure 4:**
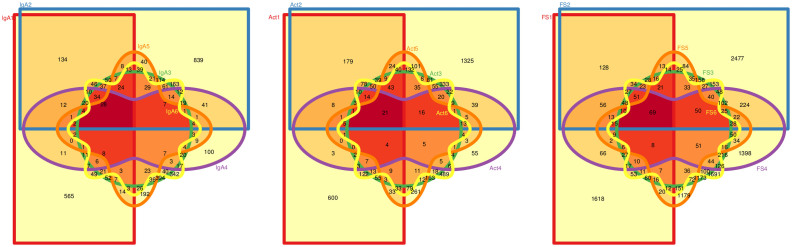
Venn diagrams of species-level OTUs^97^ distributions. Graphs show the unique and shared OTUs in FS, Act and IgA fractions, respectively. Sample code is reported close to each area. Numbers in central overlapping areas refer to the shared OTUs^97^ in the core microbiota.

Comparative analysis of species-level operational taxonomic units (sequences at 97% similarity, 80% overlapping, hereinafter OTUs^97^) revealed that among all samples, 28, 21, and 69 OTUs^97^ were shared, respectively, among IgA, Act and FS fractions (see Figure 4). The shared diversity of the FS core comprised only members of highly abundant families of *Firmicutes* and *Bacteriodetes*, while in Act and IgA fractions, members of *Proteobacteria* were also found, such as *Alphaproteobacteria* mainly belonging to *Sphingomonadales, Gammaproteobacteria* belonging to *Pseudomonadales*, and *Betaproteobacteria* belonging to *Burkholderiales*; in the IgA core we also found members of *Actinobacteria*, which were absent in the FS (see Table 2).

A less restrictive analysis describing the core bacteria made by clusters represented in at least 5 out of 6 samples showed that the FS core was almost invariable, adding only members of *Streptococcaceae*. The Act fraction also included members of *Xanthomonadaceae* from *Gammaproteobacteria*; *Microbacteriaceae* and N*ocardiaceae* from *Actinobacteria* (see Table 2).

### Taxonomy analysis

#### Unassigned genera

Genera were considered unassigned when annotated by RDP classifier with confidence value score lower than 0.8. These were annotated using upper taxonomic rank levels. In all samples a very high rate of unassigned genera was found. In FS we found that unassigned genera ranged from 24.77% of reads in V6 sample to 44.56% in V4 sample. In the Act fraction, the percentage of unidentified genera was more uniformly distributed, ranging from 8.27% (V3) to 18.83% (V4), except for sample V1 (0.46%). In S-IgA-coated fractions, unidentified genera ranged from 2.45% in V1 to 35.01% in V2.

The number of OTUs^97^ belonging to unassigned genera was of 9,122 out of 14,558. These data are shown grouped by main classes in Supplemental Information, Figure 1.

#### Firmicutes

As expected, *Clostridiales* was the most populated order not only within *Firmicutes* but also considering the rest of phyla. Overall, among taxa with frequencies equal to or greater than 1% we found: *Lachnospiraceae* (including: *Lachnospira*, *Roseoburia, Incertae Sedis XIV, Blautia*); *Ruminococcaceae* (*Faecalibacterium*) and other unidentified Clostridiales (see Supplemental Information, Figure 2 - *Firmicutes*). Within the *Bacilli* class, we found several low-frequency (less than 1% in frequency) families and genera belonging only to Act or IgA fractions. Within *Lactobacillales*, members of *Enterococcus*, some unidentified *Enterococcaceae* and *Lactobacillus* were found to be common to all samples, at least in Act and IgA fractions. Within *Streptococcaceae*, *Streptococcus* is the one shared among all samples (see Supplemental Information, Figure 2 - *Firmicutes*).

#### Bacteroidetes

*Bacteroidetes* was the second most recruited phylum, in which the genus *Bacteroides* was found among all but sample V1. It was highly recruited from the FS fraction, accounting for up to 38.86% of total reads in sample V6 (19.81%, 15.21%, 13.93%, 7.80% in V5, V3, V4 and V2 respectively). Several groups of *Bacteroidetes* were mainly recruited from the FS fractions, for example, unidentified *Porphyromonadaceae* were found uniquely in FS fractions from all but V5 samples. *Prevotella* was also one of the universally recruited groups in Act (all samples), IgA (samples V1, V2, V3 and V5) and FS (samples V1, V2, V3, V4) fractions. *Parabacteroides* was similar to *Prevotella*; it was found in FS (all but V6), Act (all samples) and IgA (all but V5) fractions (see Supplemental Information, Figure 2 - *Bacteroidetes*).

#### Proteobacteria

Almost all *Proteobacteria* have been found as part of active and/or S-IgA-opsonised microbiota.

Only *Sphingomonas*, member of *Alphaproteobacteria*, was found at frequency higher than 1% at least in the IgA fraction of V1, V2, V3 and V4; although they were also present in less than 1% in V6 (Act and IgA). *Pseudomonas* was also highly recruited in Act and IgA fractions of samples V3, V4 and V6. No *Proteobacteria* over 1% in frequency was found in sample V5. Several families of *Sphingomonadales* were found to be common to all samples, such as *Porphyrobacter* and unassigned *Erythrobacteraceae* (all but V4 and V5), *Novosphingobium* (all samples), *Sphingomonas* (all but V5) and other unidentified families (see Supplemental Information, Figure 2 - *Proteobacteria*).

Within *Betaproteobacteria*, order *Burkholderiales*, family *Sutturellaceae*, *Parasutterella* was commonly retrieved from all fractions. Also *Sutterella* from the same family was found in all but V4 and V5 samples. Within *Comamonadaceae*, *Pelomonas* was found in all samples but never in FS fractions.

Some members of *Deltaproteobacteria*, mainly related to *Desulfovibrionales* were found, but without any kind of pattern and mainly present in FS fractions.

*Gammaproteobacteria* such as *Escherichia/Shigella* were found in low frequency among all samples: in FS (V5 and V6 samples), Act (all samples) and IgA (V1, V2, V5 and V6). Other *Enterobacteriaceae* such as *Morganella* was found in the IgA fraction of all samples. Interestingly, members related to *Alcanivorax* from *Oceanospirillales*, were found in all but V4 samples at least in the IgA fraction. Within *Pseudomonadales*, *Acinetobacter* was found in all but V2 samples in Act and/or IgA fractions. *Pseudomonas* was also one of the common genera retrieved from all Act and IgA fractions, but not from FS. Also *Stenotrophomonas* from *Xanthomonadales* was found in all but V2 samples from Act or IgA (or both) fractions.

#### Actinobacteria

No genera belonging to *Actinobacteria* were found at a frequency higher than 1%. Within *Actinomycetales*, *Microbacterium* from *Microbacteriaceae* was found to be commonly present in all samples in Act (except for V1) and/or IgA but never in the FS fraction. The same trend was observed for *Rhodococcus* from *Nocardiaceae* and *Propionibacterium* from *Propionibacteriaceae*. Within *Coriobacteriales*, *Collinsella* was retrieved from all but V5 samples whereas in samples V2 and V4 it was also recruited from the FS fraction (see Supplemental Information, Figure 2 - *Actinobacteria*).

## Discussion

It is now widely accepted that most of the relationships between micro-organisms and higher organisms (e.g., plants, animals and others) are most probably commensal or mutualistic^31^. In this frame, there is growing evidence that the immune system evolved accommodating bacterial colonization of growing complexity modulating its reactivity^18^,^32^,^33^. In this work, we show that taxonomic distribution obtained from active and S-IgA-coated bacterial fractions isolated from healthy human volunteers' faecal samples are highly dissimilar from those obtained from DNA extracted directly from faecal material. Generally speaking, what we observed in FS is coherent with results obtained in several other studies concerning gut microbiota distribution, in which stool samples are used^34^. For example, there are previous reports that dominant OTUs are the ones which are more widespread among samples^23^. *Firmicutes* and *Bacteroidetes* are the main taxa commonly retrieved from all human stool samples and their ratio is often influenced by diet^35^. As also observed by Tap and collaborators^34^, several dominant taxa belonging to *Lachnospiraceae*, *Clostridiaceae* or *Bacteroidaceae* have commonly been found in the core microbiota of FS fractions of all samples, as also in this work. In addition, the use of flow cytometry-based sorting to obtain fractions, with subsequent pyrosequencing, enabled us to access active and S-IgA-coated bacterial fractions.

It may seem contradictory that many bacteria found in the active and S-IgA-coated fractions were not detected in the faecal controls. The reason for this lack of detection is probably related to the lack of amplification of rare bacteria, as PCR is biased to preferentially amplify the most common DNA templates in the sample^36^. Thus, the dominance of some bacterial taxa in faecal samples masks an important part of the diversity, which includes active and IgA-opsonised bacteria.

We found that overall the ratio of *Bacteroidetes/Firmicutes* recruitments is maintained in FS, Act and IgA microbiota but, in the latter two fractions, their presence decreased in favour of other phyla, such as *Proteobacteria* and *Actinobacteria*. Thus employing the RNA/per cell content as a measure of bacterial activity^25^,^26^, we show that several still unidentified or very rare taxa are indeed active players of human microbiota and in strict interaction with the immune system. In this context, when active and S-IgA-coated bacterial fractions were characterized, we were able to widen the microbiota core to members of *Proteobacteria* including *Sphingomonadaceae* and *Pseudomonadaceae*, while, in a less restricted core (in five out of six samples), we also found members of *Actinobacteria*. The presence of core S-IgA-coated bacteria is coherent with observations that the immune system is continuously stimulated by commensal bacteria^37^,^38^.

Within *Firmicutes*, we found that active core microbiota includes members of *Blautia* whose species *B. productia* has been tested as active fermenter in simplified human intestinal microbiota experiments using germ-free rats^39^. Other members of *Firmicutes*, such as *Lactobacillus* or *Enterococcus*, which are important due to their role as immune system modulators or for helping intestinal absorption^40^, have been commonly found as part of active and IgA fractions.

Regarding *Bacteroides*, strict anaerobes such as members of genus *Prevotella*, known as cellulose and xylane degraders, were already found as part of the core microbiota of children whose diet was high in fibre content^35^. In our experiment we found *Prevotella* as core bacteria only when we focused our attention on active or S-IgA-coated bacteria. Thus, although members of this group are considered rare, they are commonly found to be active.

A very interesting finding is the case of the phylum *Proteobacteria*, whose members are almost invisible when DNA extraction from faecal samples is used without the application of any selection or sorting protocol. In our work, we pointed out that *Proteobacteria* are recurrent active members of gut microbiota, as well as in strict interaction with the immune system. *Sphingomonadales* members (from *Alphaproteobacteria*) are important players shaping the immune system. *Sphingomonas* sp. was highly retrieved in four out of six samples while it was found in less than 1% in frequency in the other two samples. Studies in germ-free mice supplemented with conventional or restricted gut microorganisms show that glycosylceramides, which are a component of *Sphingomonas* cell wall, are identified as CD1d ligands stimulating invariant natural killer T cells^18^,^41^,^42^. We observed that several other *Sphingomonadales* families appear to be represented in the core microbiota, including the genera *Porphyrobacter*, *Novosphingobium*, *Sphingomonas* and others not well characterized yet but falling within the *Sphingomonadaceae* family.

Other commonly retrieved *Proteobacteria* retrieved from all fractions were members of Beta class, belonging to the order *Burkholderiales*. *Parasutturella* and *Sutturella* have been linked to healthy flora in studies of lean and obese mice^43^. *Burkholderia* were also commonly retrieved from active and S-IgA-coated microbial fractions but never in FS fraction probably due to their reduced frequency overwhelmed by dominant bacteria when a standard stool-based approach is applied. *Burkholderia* is often considered to be an opportunistic pathogenic bacteria but its commensal role makes it apt to survive in the intestine without generating infection processes. Hutchinson and collaborators reported *Burkholderia* as strict aerobic bacteria although viable colonies have also been reported to appear after days of anaerobic incubation^44^. This could explain its scarce presence and recruitment from the gastrointestinal tract in active and S-IgA-coated fractions.

*Escherichia/Shigella* related *Enterobacteriaceae* were found in almost all samples and fractions. We also found *Alcanivorax*-related members, which are known to be mostly marine, active obligate oil-degrading bacteria^45^. Although its 16S rDNA homology with the closest member in GenBank is around 96% (data not shown), it is surprising to find it in active or S-IgA-coated fractions of almost all samples. Another *Gammaproteobacteria* identified was *Acinetobacter* (family *Moraxellaceae*) although its 16S similarity is far from *A. baumani* (97% similarity) and closer (99%) to other unidentified or uncultured *Acinetobacter* (data not shown). In a recent study in mice *Acinetobacter*, *Stenotrophomonas* and *Comamonas* are hypothesized to be part of the so-called “crypt-specific core microbiota”^46^. Here we found that these groups of bacteria are almost always recruited from human active microbiota or from human S-IgA-coated microbiota. Some genera were commonly found in Act and IgA fractions but they seem to be hidden when considering FS fractions.

Moreover, *Actinobacteria* were commonly recruited at frequencies lower than 1%. In their review, Turroni and collaborators^47^ described *Actinobacteria* to have been commonly retrieved from faecal samples using classical culture-dependent approaches, but they are rarely found in metagenomics-based experiments^2^,^48^,^49^. Likewise, in our samples, *Actinobacteria* were almost absent in FS fractions while they were low-frequency recruited in active and/or S-IgA-coated bacterial fractions. Thus, their absence in the FS fraction cannot be attributed to some biases in the DNA extraction method (commonly applied to all fractions) or to the primers used for PCR amplification, but rather a result of the sequencing optimization when a specific fraction is selected using the flow-cytometry-based sorting approach.

In conclusion, the proposed approach enabled us to obtain 16S rDNA amplicons from genomic DNA of active bacterial cells, selected and sorted by flow cytometry. Compared to other methodologies where active bacteria are identified by retrotranscription of ribosomal RNA, the taxonomic distributions observed in this work are not biased by the rRNA copy number, found in each cell, while it is possibly biased only by the chromosomal 16S rDNA copies (chromosomal 16S rDNA). As already pointed out in our previous work^21^, the active fraction of gut microbiota represents a different approach to studying the human microbiota. The combination of this approach with the characterization of bacteria opsonised by different human immunoglobulins will allow future study of the active bacteria ignored by the immune system, as well as those actively growing but opsonised by different Ig types. We believe this will provide important insights into the host-microbiota interplay under both healthy and disease-related conditions.

Finally, we describe the presence of a microbiota core among faecal samples of six healthy volunteer samples. This core contains members of taxa already described in previous works; however, focusing the analysis on fractions of active or S-IgA-coated bacteria highlights the presence of other “important” taxa, which were previously not visible. For instance, we found that in addition to the already known members of the gut microbiota core, such as *Firmicutes* and *Bacteroidetes*, other active bacteria such as members of *Actinobacteria* and *Proteobacteria* (including genera of the *Sphingomonadaceae* and *Moraxellaceae* families) may play an important role in gut homeostasis that has yet to be elucidated.

## Methods

### Samples and fractions

Samples were obtained from six healthy volunteers (between 20 and 36 years old, three male and three female) identified as V1, V2, V3, V4, V5 and V6. All participants expressed their interest in participating in this study by signing an informed consent form, approved by the Ethics and Research Committee of Centre for Public Health Research (CSISP) of Valencia, Spain. One of the main conditions of exclusion was that no antibiotics had been administrated during the last two months prior to sampling. None of the volunteers had organic intestinal disorders. All volunteers follow a Mediterranean diet.

For each faecal sample, we studied the taxonomical distribution of faecal suspension (FS), active bacteria (Act) and S-IgA-coated bacterial fractions (IgA).

The volunteers collected faecal material in sterile 30 ml screw-cap containers (25690 mm; PP SPOON; DELTALAB), containing 8 ml RNAlater (Ambion #AM7020) in order to preserve RNA. Samples were kept at −25°C, delivered to the lab within the next 24 hours and immediately processed. From each sample, the faecal material was resuspended by vortexing (2 min). It was washed twice in physiological solution (NaCl 0.9%). Faecal suspension was centrifuged (800 g) for 2 min to pellet big aggregates. Then, supernatant was centrifuged at 7500 g for 7 min to collect microbial cells from faecal suspension. Pellet was washed twice in physiological solution (NaCl 0.9%). Cells were immediately fixed adding 1/10 volume of 37% formaldehyde (final concentration: 3.7%) and incubated over-night at 4°C. Fixed cells were washed twice to remove residual formaldehyde and resuspended in 0.1 ml of physiological solution. The samples were stored at −20°C after adding 1 volume of absolute ethanol. Three fractions were obtained from each sample stored in the previous step. The DNA in the first aliquot was extracted prior to flow cytometry steps (FS fraction). The second and third fractions were obtained by flow cytometry sorting. Thus we obtained: the active population by the mean of RNA content and the S-IgA-coated population using anti-human IgA immunoglobulin. For staining and flow cytometry protocols, fixed cells from previous steps were washed and diluted to achieve an optical density (O.D. 600) around 0.2 using physiological solution.

### Staining

#### Cell labelling and flow cytometry sorting

Before sorting, cell suspensions were disaggregated by mild sonication and filtering, so cells ran freely in the microfluidic system (see SI Figure 3). Ten microlitres of pyronin-Y (Sigma-Aldrich, #P9172, 10 mg/ml) diluted to 100 μM was added to the samples (1 ml of volume) for total RNA staining and incubated for 1 hour at 4°C. The samples were then stained with SYTO62 (Invitrogen, #S11344) according to manufacturer instructions (final concentration of 0.5 μM) in order to distinguish the bacteria from the noise during the flow cytometry, by the mean of their DNA content. S-IgA-coated bacterial staining was performed using anti-human IgA labelled with FITC (Invitrogen, #62-7411). Anti-mouse IgA labelled with FITC (Invitrogen, #M31001) was used for isotype control (see Supplemental Information, Figures 3 and 4).

Flow cytometry sorting was carried out using the MoFlo™ XDP Cell Sorter. The light sources were the Argon 488 nm (blue) laser (200 mW power) and the 635 nm (red) diode laser (25 mW power). The lasers were aligned using *Flow-Check™* (10 μ*m*) and *Flow-Set™* (3 μ*m*). The cytometer emission filter was 520/30 (FL1), 580/30 (FL2) and 680/30 (FL4) obtaining emission for FITC, Pyronin Y and SYTO62, respectively. The trigger was set on side-scatter.

### DNA extraction and sequencing

DNA extraction from all fractions was carried out using the CTAB method^50^. Total 16S rDNA was amplified from each fraction using 8F and 530R universal primers for bacteria^51^ using multiplex identifiers (MIDs, Supplemental Information, Table 2). PCR products obtained from each fraction/sample were purified by Nucleofast 96 PCR filter plates (Macherey Nagel #74310050) and concentrations were measured by PicoGreen assay. The products were pooled obtaining balanced final concentrations and sequenced using the 454 GS-FLX pyrosequencer (Titanium chemistry, Roche).

### Bioinformatics

Obtained sequences were trimmed from the end in sliding windows of 10 nucleotides when the average quality value was lower than 20, using Prinseq (v0.19.4) software^52^. This considerably improved the quality of the reads, as the quality of pyrosequencing reads has been shown to dramatically decrease towards the end of the sequences^53^. All sequences shorter than 200 nucleotides were not considered. Taxonomic assignations were carried out using the RDP_classifier^54^, and phylogenetic ranks were assigned when scores exceeded 0.8. Clustering was performed by the use of CD-HIT software^55^.

Percentage distributions of fractions coming from flow cytometry sorting (Act and IgA) were multiplied by the cell abundance rate of the sorted fraction with respect to the total amount of cells counted during the whole sorting (see Table 1 column “Fraction rate”). Percentage values of FS fractions remained unaltered.

Descriptive and statistical analyses were carried out with the R statistic environment using Vegan R package^56^,^57^. Venn diagrams for pan-microbiota analysis were obtained using Vennerable R package^58^. Flow cytometry data were analysed using R package flowCore and flowViz from Bioconductor^59^,^60^,^61^.

### Accession numbers

Sequences were deposited in EMBL-EBI Sequence Read Archive (SRA) under study number ERP002046.

## Author Contributions

 G.D., F.P.B. and A.Mo Conceived and designed the experiments. G.D., F.P.B., and M.D. performed the experiments. G.D., F.P.B., A.Mi. and M.C.C. analyzed the data. G.D., F.P.B., A.L., A.Mi. and A.Mo. wrote the main manuscript

## Supplementary Material

Supplementary Information Supplemental Information

## Figures and Tables

**Table 1:** Details of samples/fractions. For each sample and fraction columns describe for each sample and fraction, the number of sorted cells, the proportion of sorted cells with respect to total number, the number of genera, the Shannon index of diversity, the Chao1 richness and its standard deviation, respectively

Sample	Fraction	Sorted cells	Fraction rate	Reads	Genera	Shannon	Chao1	SE.Chao1
V1	FS	NA*	1	6636	77	3.02	89.75	9.09
	IgAPos	51010	0.1	4483	101	3.56	112.77	8.32
	PYPos	28052	0.06	14359	107	2.47	120	11.71
V2	FS	NA	1	10327	74	2.59	97.75	17.56
	IgAPos	217181	0.8	6653	96	3.02	113	12.75
	PYPos	142527	0.34	13222	104	2.8	112.08	6.34
V3	FS	NA	1	10869	70	2.24	83.91	10.04
	IgAPos	206999	0.98	3248	92	2.82	109.25	10.26
	PYPos	34087	0.45	2105	92	3.24	101.71	7.05
V4	FS	NA	1	9922	66	2.23	83.5	11.54
	IgAPos	19745	0.21	974	69	3.17	81.21	8.32
	PYPos	206352	0.66	699	41	2.5	44.5	4.84
V5	FS	NA	1	10205	41	1.88	50.33	16.49
	IgAPos	94701	0.32	1612	46	2.33	61.6	16.43
	PYPos	242980	0.61	4348	60	1.98	81	20.9
V6	FS	NA	1	11737	50	2.17	72.5	59.58
	IgAPos	21672	0.57	2657	78	3.02	91.6	10.26
	PYPos	130000	0.41	9084	87	2.82	92.14	5.88

*DNA from FS fractions was obtained directly from faecal suspension without flow cytometry steps, see Materials and Methods.

**Table 2:** Core microbiota. Columns describe taxonomic ranks of core OTUs^97^ of each fraction among all samples. Asterisks indicate families found in at least 5 out of 6 samples

Fraction	Phylum	Class	Order	Family
FS	Bacteroidetes	Bacteroidia	Bacteroidales	Bacteroidaceae
	Firmicutes	Clostridia	Clostridiales	Lachnospiraceae
				Ruminococcaceae
				ud-Clostridiales
		Bacilli	Lactobacillales	Streptococcaceae*
	ud-Bacteria	ud-Bacteria	ud-Bacteria	ud-Bacteria
Act	Actinobacteria	Actinobacteria	Actinomycetales	Microbacteriaceae*
				Nocardiaceae*
	Bacteroidetes	Bacteroidia	Bacteroidales	Bacteroidaceae
	Proteobacteria	Alphaproteobacteria	Sphingomonadales	Erythrobacteraceae
				Sphingomonadaceae
				ud-Sphingomonadales
		Betaproteobacteria	Burkholderiales	Comamonadaceae
		Gammaproteobacteria	Pseudomonadales	Pseudomonadaceae
			Xanthomonadales	Xanthomonadaceae*
	Firmicutes	Clostridia	Clostridiales	Ruminococcaceae*
				Lachnospiraceae
				ud-Clostridiales
		Bacilli	Lactobacillales	Streptococcaceae*
IgA	Actinobacteria	Actinobacteria	Actinomycetales	Nocardiaceae
	Bacteroidetes	Bacteroidia	Bacteroidales	Bacteroidaceae
	Proteobacteria	Alphaproteobacteria	Sphingomonadales	Sphingomonadaceae
				ud-Sphingomonadales
		Betaproteobacteria	Burkholderiales	Comamonadaceae
		Gammaproteobacteria	Pseudomonadales	Pseudomonadaceae
	Firmicutes	Bacilli	Lactobacillales	Enterococcaceae
		Clostridia	Clostridiales	Ruminococcaceae
				Lachnospiraceae
		Bacilli	Lactobacillales	Streptococcaceae*

## References

[b1] Stark P. L. & Lee A. The microbial ecology of the large bowel of breast-fed and formula-fed infants during the first year of life. J Med Microbiol 15, 189–203 (1982). 7143428 10.1099/00222615-15-2-189

[b2] Eckburg P. B. *et al.* Diversity of the human intestinal microbial flora. Science (New York, N.Y.) 308, 1635–8 (2005). 10.1126/science.1110591 PMC1395357 15831718

[b3] Gill S. R. *et al.* Metagenomic Analysis of the Human. 1355, (2007). 10.1126/science.1124234 PMC3027896 16741115

[b4] Qin J. *et al.* A human gut microbial gene catalogue established by metagenomic sequencing. Nature 464, 59–65 (2010). 20203603 10.1038/nature08821 PMC3779803

[b5] Favier C. F., Vaughan E. E., De Vos W. M. & Akkermans A. D. L. Molecular monitoring of succession of bacterial communities in human neonates. Appl Environ Microbiol 68, 219–26 (2002). 11772630 10.1128/AEM.68.1.219-226.2002 PMC126580

[b6] Penders J. *et al.* Factors influencing the composition of the intestinal microbiota in early infancy. Pediatrics 118, 511–21 (2006). 16882802 10.1542/peds.2005-2824

[b7] Bezirtzoglou E. The intestinal microflora during the first weeks of life. Anaerobe 3, 173–7 (1997). 16887585 10.1006/anae.1997.0102

[b8] Mulder I. E. *et al.* Environmentally-acquired bacteria influence microbial diversity and natural innate immune responses at gut surfaces. BMC Biol 7, 79 (2009). 19930542 10.1186/1741-7007-7-79 PMC2785767

[b9] Vaishampayan P. A. *et al.* Comparative metagenomics and population dynamics of the gut microbiota in mother and infant. Genome Biol Evol 2, 53–66 (2010). 20333224 10.1093/gbe/evp057 PMC2839348

[b10] Mändar R., Mikelsaar M. & Mandar R. Transmission of mother's microflora to the newborn at birth. Biology of the neonate 69, 30–5 (1996). 8777246 10.1159/000244275

[b11] Dethlefsen L., McFall-Ngai M. & Relman D. A. An ecological and evolutionary perspective on human-microbe mutualism and disease. Nature 449, 811–8 (2007). 17943117 10.1038/nature06245 PMC9464033

[b12] Buhnik-Rosenblau K. *et al.* Indication for Co-evolution of Lactobacillus johnsonii with its hosts. BMC microbiology 12, 149 (2012). 22827843 10.1186/1471-2180-12-149 PMC3503616

[b13] McFall-Ngai M. Adaptive immunity: care for the community. Nature 445, 153 (2007). 17215830 10.1038/445153a

[b14] Ley R. E., Peterson D. a. & Gordon J. I. Ecological and evolutionary forces shaping microbial diversity in the human intestine. Cell 124, 837–48 (2006). 16497592 10.1016/j.cell.2006.02.017

[b15] Medzhitov R. Toll-like receptors and innate immunity. Nat Rev Immunol 1, 135–45 (2001). 11905821 10.1038/35100529

[b16] Lotz M. *et al.* Postnatal acquisition of endotoxin tolerance in intestinal epithelial cells. J Exp Med 203, 973–84 (2006). 16606665 10.1084/jem.20050625 PMC2118301

[b17] Giraud A. *et al.* Dissecting the genetic components of adaptation of Escherichia coli to the mouse gut. PLoS genetics 4, e2 (2008). 18193944 10.1371/journal.pgen.0040002 PMC2174974

[b18] Wei B. *et al.* Commensal microbiota and CD8+ T cells shape the formation of invariant NKT cells. Journal of immunology (Baltimore, Md.: 1950) 184, 1218–26 (2010). 10.4049/jimmunol.0902620 PMC3458428 20048124

[b19] Conley M. E. & Delacroix D. L. Intravascular and mucosal immunoglobulin A: two separate but related systems of immune defense? Annals of internal medicine 106, 892–9 (1987). 3579073 10.7326/0003-4819-106-6-892

[b20] Van der Waaij L. a. *et al.* Immunoglobulin coating of faecal bacteria in inflammatory bowel disease. European Journal of Gastroenterology & Hepatology 16, 669–674 (2004). 15201580 10.1097/01.meg.0000108346.41221.19

[b21] Peris-Bondia F., Latorre A., Artacho A., Moya A. & D'Auria G. The active human gut microbiota differs from the total microbiota. PloS one 6, e22448 (2011). 21829462 10.1371/journal.pone.0022448 PMC3145646

[b22] Wylie K. M. *et al.* Novel bacterial taxa in the human microbiome. PloS one 7, e35294 (2012). 22719826 10.1371/journal.pone.0035294 PMC3374617

[b23] Huse S. M., Ye Y., Zhou Y. & Fodor A. A. A Core Human Microbiome as Viewed through 16S rRNA Sequence Clusters. PLoS one 7, 1–12 (2012). 10.1371/journal.pone.0034242 PMC3374614 22719824

[b24] van der Waaij L. A., Mesander G., Limburg P. C. & van der Waaij D. Direct flow cytometry of anaerobic bacteria in human feces. Cytometry 16(3), 270–9 (1996). 7924697 10.1002/cyto.990160312

[b25] Amann R., Ludwig W. & Schleifer K. Phylogenetic identification and in situ detection of individual microbial cells without cultivation. Microbiological reviews 59, 143–169 (1995). 7535888 10.1128/mr.59.1.143-169.1995 PMC239358

[b26] Poulsen L. K., Ballard G. & Stahl D. a. Use of rRNA fluorescence in situ hybridization for measuring the activity of single cells in young and established biofilms. Applied and environmental microbiology 59, 1354–60 (1993). 7685999 10.1128/aem.59.5.1354-1360.1993 PMC182089

[b27] Anderson M. J. A new method for non-parametric multivariate analysis of variance. Austral Ecology 26, 32–46 (2001).

[b28] McArdle B. H. & Anderson M. J. Fitting multivariate models to community data: A comment on distance-based redundancy analysis. Ecology 82, 290–297 (2001).

[b29] Mira A., Martín-Cuadrado A. B., D'Auria G. & Rodríguez-Valera F. The bacterial pan-genome:a new paradigm in microbiology. International microbiology: the official journal of the Spanish Society for Microbiology 13, 45–57 (2010). 20890839 10.2436/20.1501.01.110

[b30] Tettelin H. *et al.* Genome analysis of multiple pathogenic isolates of Streptococcus agalactiae: implications for the microbial “pan-genome”. Proceedings of the National Academy of Sciences of the United States of America 102, 13950–13955 (2005). 16172379 10.1073/pnas.0506758102 PMC1216834

[b31] Moya A., Peretó J., Gil R. & Latorre A. Learning how to live together: genomic insights into prokaryote-animal symbioses. Nature reviews. Genetics 9, 218–29 (2008). 10.1038/nrg2319 18268509

[b32] Cerf-Bensussan N. & Gaboriau-Routhiau V. V. The immune system and the gut microbiota: friends or foes? Nature Reviews Immunology 10, 735–744 (2010). 10.1038/nri2850 20865020

[b33] Moran N. A., McCutcheon J. P. & Nakabachi A. Genomics and evolution of heritable bacterial symbionts. Annual review of genetics 42, 165–90 (2008). 10.1146/annurev.genet.41.110306.130119 18983256

[b34] Tap J. *et al.* Towards the human intestinal microbiota phylogenetic core. Environmental microbiology 11, 2574–2584 (2009). 19601958 10.1111/j.1462-2920.2009.01982.x

[b35] De Filippo C. *et al.* Impact of diet in shaping gut microbiota revealed by a comparative study in children from Europe and rural Africa. Proceedings of the National Academy of Sciences of the United States of America 107, 14691–6 (2010). 20679230 10.1073/pnas.1005963107 PMC2930426

[b36] Gonzalez J. M., Portillo M. C., Belda-ferre P. & Mira A. Amplification by PCR Artificially Reduces the Proportion of the Rare Biosphere in Microbial Communities. 7, (2012). 10.1371/journal.pone.0029973 PMC3256211 22253843

[b37] Collier-Hyams L. S. & Neish A. S. Innate immune relationship between commensal flora and the mammalian intestinal epithelium. Cellular and molecular life sciences: CMLS 62, 1339–48 (2005). 15971109 10.1007/s00018-005-5038-y PMC11924482

[b38] Majamaa H., Isolauri E., Saxelin M. & Vesikari T. Lactic acid bacteria in the treatment of acute rotavirus gastroenteritis. Journal of pediatric gastroenterology and nutrition 20, 333–8 (1995). 7608829 10.1097/00005176-199504000-00012

[b39] Becker N., Kunath J., Loh G. & Blaut M. Human intestinal microbiota: characterization of a simplified and stable gnotobiotic rat model. Gut microbes 2, 25–33. 21637015 10.4161/gmic.2.1.14651

[b40] Vizoso Pinto M. G. *et al.* Lactobacilli stimulate the innate immune response and modulate the TLR expression of HT29 intestinal epithelial cells in vitro. International journal of food microbiology 133, 86–93 (2009). 19523707 10.1016/j.ijfoodmicro.2009.05.013

[b41] Mattner J. *et al.* Exogenous and endogenous glycolipid antigens activate NKT cells during microbial infections. Nature 434, 525–9 (2005). 15791258 10.1038/nature03408

[b42] Kinjo Y. *et al.* Recognition of bacterial glycosphingolipids by natural killer T cells. Nature 434, 520–5 (2005). 15791257 10.1038/nature03407

[b43] Murphy E. F. *et al.* Divergent metabolic outcomes arising from targeted manipulation of the gut microbiota in diet-induced obesity. Gut (2012). 10.1136/gutjnl-2011-300705. 10.1136/gutjnl-2011-300705 22345653

[b44] Hutchison M. L., Poxton I. R. & Govan J. R. Burkholderia cepacia produces a hemolysin that is capable of inducing apoptosis and degranulation of mammalian phagocytes. Infection and immunity 66, 2033–9 (1998). 9573086 10.1128/iai.66.5.2033-2039.1998 PMC108160

[b45] Yakimov M. M., Timmis K. N. & Golyshin P. N. Obligate oil-degrading marine bacteria. Current opinion in biotechnology 18, 257–66 (2007). 17493798 10.1016/j.copbio.2007.04.006

[b46] Pédron T. *et al.* A crypt-specific core microbiota resides in the mouse colon. mBio 3, (2012). 10.1128/mBio.00116-12 PMC3372965 22617141

[b47] Turroni F., Ribbera A., Foroni E., Van Sinderen D. & Ventura M. Human gut microbiota and bifidobacteria: from composition to functionality. Antonie van Leeuwenhoek 94, 35–50 (2008). 18338233 10.1007/s10482-008-9232-4

[b48] Palmer C., Bik E. M., DiGiulio D. B., Relman D. A. & Brown P. O. Development of the human infant intestinal microbiota. PLoS Biol 5, e177 (2007). 17594176 10.1371/journal.pbio.0050177 PMC1896187

[b49] Suau A. *et al.* Direct analysis of genes encoding 16S rRNA from complex communities reveals many novel molecular species within the human gut. Applied and environmental microbiology 65, 4799–807 (1999). 10543789 10.1128/aem.65.11.4799-4807.1999 PMC91647

[b50] Ausubel F. M. *et al.* Current protocol in molecular biology. Current protocols in molecular biology 211–245 (Greene, Publishing Association; Wiley-Interscience: New York, 1992).

[b51] Lane D. D. J., Stackenbrandt E. & Goodfellow M. 16S/23S rRNA sequencing. Nucleic Acid Techniques in Bacterial Systematics 115–175 (1991).

[b52] Schmieder R. & Edwards R. Quality control and preprocessing of metagenomic datasets. Bioinformatics (Oxford, England) 27, 863–4 (2011). 10.1093/bioinformatics/btr026 PMC3051327 21278185

[b53] Claesson M. J. *et al.* Composition, variability, and temporal stability of the intestinal microbiota of the elderly. Proceedings of the National Academy of Sciences of the United States of America 108 Suppl, 4586–91 (2011). 20571116 10.1073/pnas.1000097107 PMC3063589

[b54] Wang Q., Garrity G. M., Tiedje J. M. & Cole J. R. Naive Bayesian classifier for rapid assignment of rRNA sequences into the new bacterial taxonomy. Applied and environmental microbiology 73, 5261–7 (2007). 17586664 10.1128/AEM.00062-07 PMC1950982

[b55] Li W. & Godzik A. Cd-hit: a fast program for clustering and comparing large sets of protein or nucleotide sequences. Bioinformatics (Oxford, England) 22, 1658–1659 (2006). 10.1093/bioinformatics/btl158 16731699

[b56] Oksanen J. *et al.* vegan: Community Ecology Package. (2011). at <http://cran.r-project.org/package=vegan>.

[b57] R Development Core Team R: A Language and Environment for Statistical Computing. (2010). at <http://www.r-project.org/>.

[b58] Swinton J. Vennerable: Venn and Euler area-proportional diagrams. at <http://r-forge.r-project.org/projects/vennerable/>.

[b59] Gentleman R. C. *et al.* Bioconductor: open software development for computational biology and bioinformatics. Genome biology 5, R80 (2004). 15461798 10.1186/gb-2004-5-10-r80 PMC545600

[b60] Ellis B., Haaland P., Hahne F., Le Meur N. & Gopalakrishnan N. flowCore: Basic structures for flow cytometry data.

[b61] Ellis B., Gentleman R., Hahne F., Le Meur N. & Sarkar D. flowViz: Visualization for flow cytometry.

