# Supplementary material for: Active and secreted IgA-coated bacterial fractions from the human gut reveal an under-represented microbiota core
Source: Sci Rep. 2013 Dec 17;3:3515. doi: 10.1038/srep03515 (PMC3865468; doi:10.1038/srep03515)

Supplemental information

Title.

Active and secreted IgA-coated bacterial fractions from the human gut reveal an under-represented microbiota core.

Authors.

Giuseppe D'Auria, Francesc Peris-Bondia, Mária Džunková, Alex Mira, Maria Carmen Collado, Amparo Latorre and Andrés Moya

Table 1: Analysis of variance using distance matrix (Adonis). The F-test is based on sequential sums of squares from permutations of the raw data, and not permutations of residuals. Analysis was carried out using environmental metadata grouping data by fractions (FS, Act and IgA) and samples (V1, V2, V3, V4, V5 and V6).

	df	Sums of squares	Mean square	F statistic	R2	Pr(>F)
FRACTION	2	1.08	0.54	4.26	0.28	0.0020**
SAMPLE	5	1.58	0.32	2.49	0.40	0.0040**
Residuals	10	1.27	0.13		0.32	
Total	17	3.93			1.00	

Table 2: Multiplex identifiers to tag each PCR reaction. Same MIDs have been used in different pyrosequencing runs.

MID	Sequence	SAMPLE
MID1	TCGCTTCCTATC	V1.Iga, V1.Act, V1.Fs
MID2	TCTAATGCGCTC	V2.IgA, V2.Act, V2.FS
MID3	TCGCTGCTTGTC	V3.IgA, V3.Act, V3.FS
MID4	TCTACGAACCTC	V4.IgA, V4.Act, V4.FS
MID5	TCGGATAACGTC	V5.IgA, V5.Act, V5.FS
MID6	TCTAGCGGAATC	V6.IgA, V6.Act, V6.FS

Figure 1: All/unidentified genus distribution. Bar charts show the distributions of all and unidentified genera (as sum) among all samples and fractions. Blue gradients describe all genus frequencies within phyla. Red gradients show the proportion of unidentified genera. Data are represented in logarithmic scale. Groups of bar charts describe distributions among phyla (X axis). Each group describes distributions among fractions FS, Act and IgA respectively (see legend on top). Only major phyla are represented.

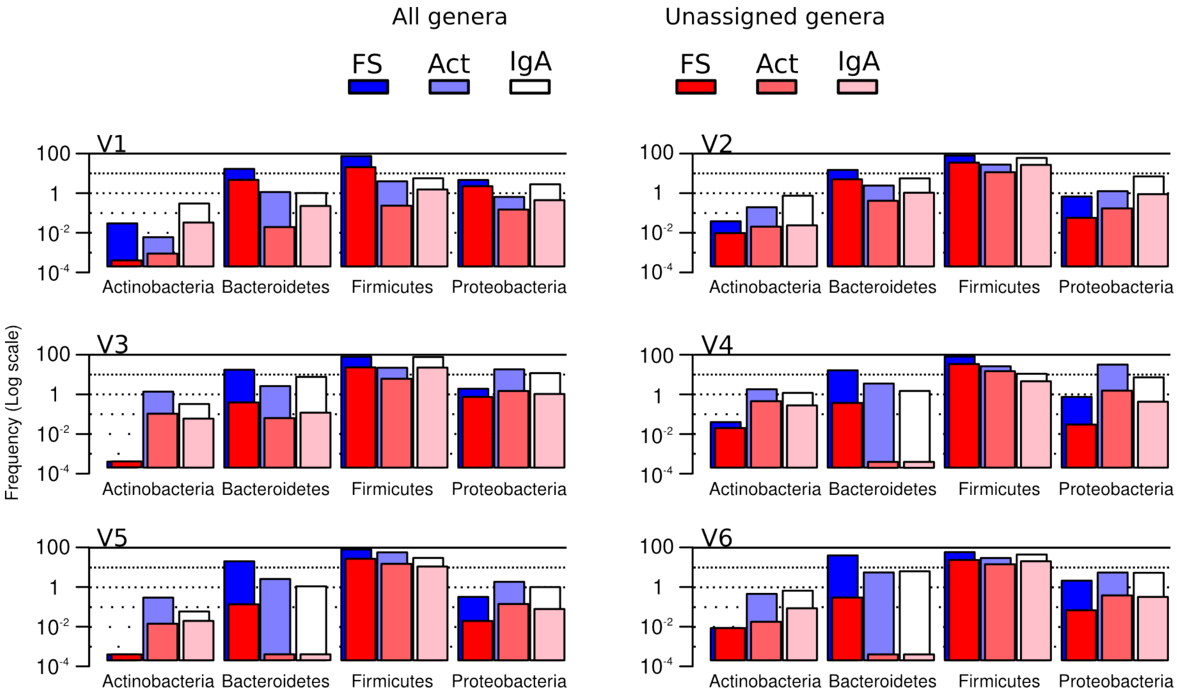

Figure 2: Bar charts describe the distribution of reads at genus level for main phyla. For each phylum every chart refers to a sample (headers). Bars represent frequency values expressed in percentage. Act and IgA values have been corrected taking into account the representativeness of the fraction with respect to the total number of cells (see Materials and Methods section). Y axis data are reported in percentage and on a logarithmic scale in order to highlight under-represented bacteria.

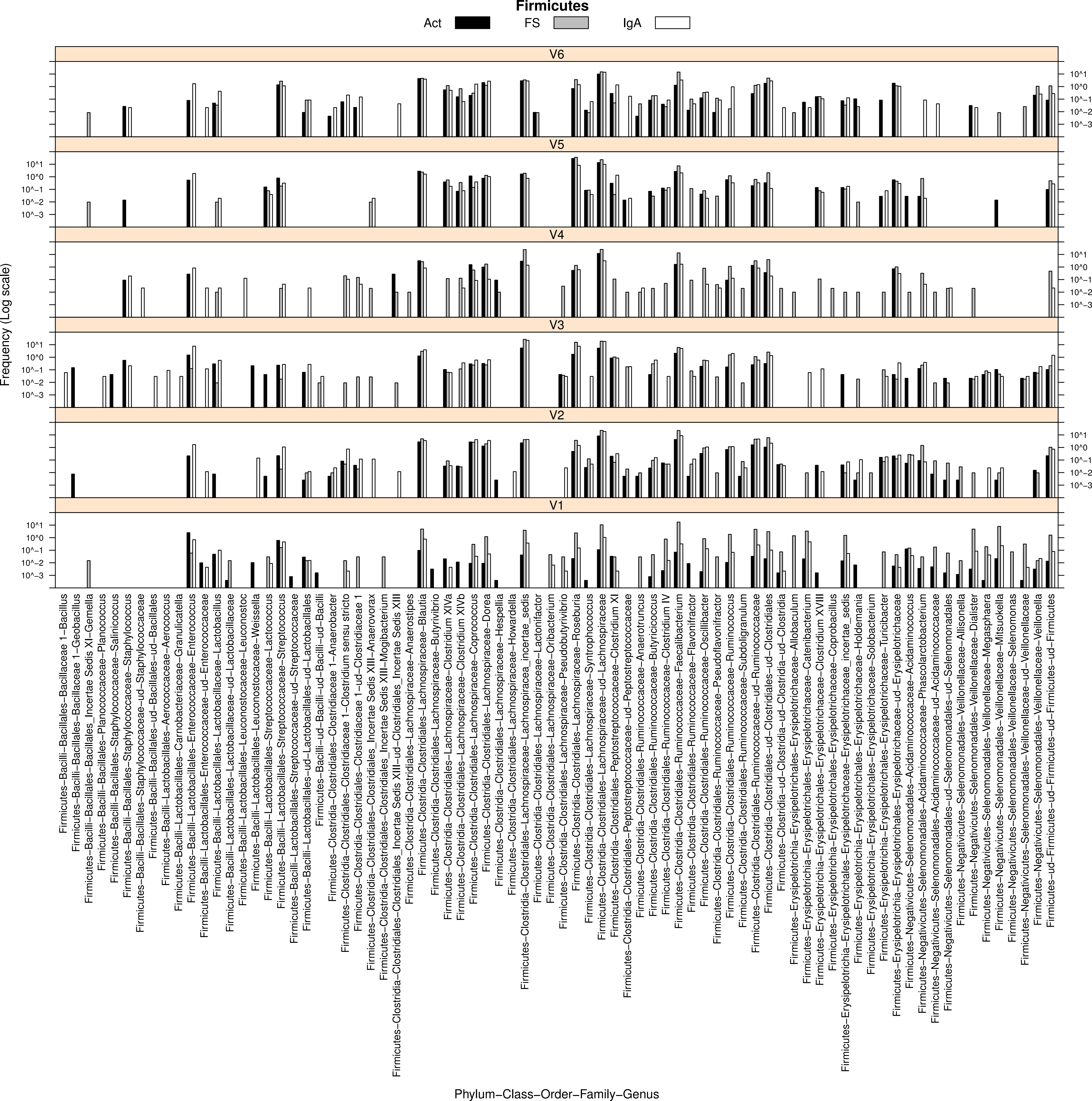

Firmicutes
Act
FS
IgA
V6
V5
V4
V3
V2
V1
10^1
10^0
10^−1
10^−2
10^−3
Frequency (Log scale)
Firmicutes–Bacilli–Bacillales–Bacillaceae 1–Bacillus
Firmicutes–Bacilli–Bacillales–Bacillaceae 1–Geobacillus
Firmicutes–Bacilli–Bacillales–Bacillales_Incertae Sedis XI–Gemella
Firmicutes–Bacilli–Bacillales–Planococcaceae–Planococcus
Firmicutes–Bacilli–Bacillales–Staphylococcaceae–Salinicoccus
Firmicutes–Bacilli–Bacillales–Staphylococcaceae–Staphylococcus
Firmicutes–Bacilli–Bacillales–Staphylococcaceae–ud–Staphylococcaceae
Firmicutes–Bacilli–Bacillales–ud–Bacillales–ud–Bacillales
Firmicutes–Bacilli–Lactobacillales–Aerococcaceae–Aerococcus
Firmicutes–Bacilli–Lactobacillales–Carnobacteriaceae–Granulicatella
Firmicutes–Bacilli–Lactobacillales–Enterococcaceae–Enterococcus
Firmicutes–Bacilli–Lactobacillales–Enterococcaceae–ud–Enterococcaceae
Firmicutes–Bacilli–Lactobacillales–Lactobacillaceae–Lactobacillus
Firmicutes–Bacilli–Lactobacillales–Lactobacillaceae–ud–Lactobacillaceae
Firmicutes–Bacilli–Lactobacillales–Leuconostocaceae–Leuconostoc
Firmicutes–Bacilli–Lactobacillales–Leuconostocaceae–Weissella
Firmicutes–Bacilli–Lactobacillales–Streptococcaceae–Lactococcus
Firmicutes–Bacilli–Lactobacillales–Streptococcaceae–Streptococcus
Firmicutes–Bacilli–Lactobacillales–Streptococcaceae–ud–Streptococcaceae
Firmicutes–Bacilli–Lactobacillales–ud–Lactobacillales–ud–Lactobacillales
Firmicutes–Bacilli–ud–Bacilli–ud–Bacilli–ud–Bacilli
Firmicutes–Clostridia–Clostridiales–Clostridiaceae 1–Anaerobacter
Firmicutes–Clostridia–Clostridiales–Clostridiaceae 1–Clostridium sensu stricto
Firmicutes–Clostridia–Clostridiales–Clostridiaceae 1–ud–Clostridiaceae 1
Firmicutes–Clostridia–Clostridiales–Clostridiales_Incertae Sedis XIII–Anaerovorax
Firmicutes–Clostridia–Clostridiales–Clostridiales_Incertae Sedis XIII–Mogibacterium
Firmicutes–Clostridia–Clostridiales–Clostridiales_Incertae Sedis XIII–ud–Clostridiales_Incertae Sedis XIII
Firmicutes–Clostridia–Clostridiales–Lachnospiraceae–Anaerostipes
Firmicutes–Clostridia–Clostridiales–Lachnospiraceae–Blautia
Firmicutes–Clostridia–Clostridiales–Lachnospiraceae–Butyrivibrio
Firmicutes–Clostridia–Clostridiales–Lachnospiraceae–Clostridium XlVa
Firmicutes–Clostridia–Clostridiales–Lachnospiraceae–Clostridium XlVb
Firmicutes–Clostridia–Clostridiales–Lachnospiraceae–Coprococcus
Firmicutes–Clostridia–Clostridiales–Lachnospiraceae–Dorea
Firmicutes–Clostridia–Clostridiales–Lachnospiraceae–Hespellia
Firmicutes–Clostridia–Clostridiales–Lachnospiraceae–Howardella
Firmicutes–Clostridia–Clostridiales–Lachnospiraceae–Lachnospiracea_incertae_sedis
Firmicutes–Clostridia–Clostridiales–Lachnospiraceae–Lactonifactor
Firmicutes–Clostridia–Clostridiales–Lachnospiraceae–Oribacterium
Firmicutes–Clostridia–Clostridiales–Lachnospiraceae–Pseudobutyrivibrio
Firmicutes–Clostridia–Clostridiales–Lachnospiraceae–Roseburia
Firmicutes–Clostridia–Clostridiales–Lachnospiraceae–Syntrophococcus
Firmicutes–Clostridia–Clostridiales–Lachnospiraceae–ud–Lachnospiraceae
Firmicutes–Clostridia–Clostridiales–Peptostreptococcaceae–Clostridium XI
Firmicutes–Clostridia–Clostridiales–Peptostreptococcaceae–ud–Peptostreptococcaceae
Firmicutes–Clostridia–Clostridiales–Ruminococcaceae–Anaerotruncus
Firmicutes–Clostridia–Clostridiales–Ruminococcaceae–Butyricicoccus
Firmicutes–Clostridia–Clostridiales–Ruminococcaceae–Clostridium IV
Firmicutes–Clostridia–Clostridiales–Ruminococcaceae–Faecalibacterium
Firmicutes–Clostridia–Clostridiales–Ruminococcaceae–Flavonifractor
Firmicutes–Clostridia–Clostridiales–Ruminococcaceae–Oscillibacter
Firmicutes–Clostridia–Clostridiales–Ruminococcaceae–Pseudoflavonifractor
Firmicutes–Clostridia–Clostridiales–Ruminococcaceae–Ruminococcus
Firmicutes–Clostridia–Clostridiales–Ruminococcaceae–Subdoligranulum
Firmicutes–Clostridia–Clostridiales–Ruminococcaceae–ud–Ruminococcaceae
Firmicutes–Clostridia–Clostridiales–ud–Clostridiales–ud–Clostridiales
Firmicutes–Clostridia–ud–Clostridia–ud–Clostridia–ud–Clostridia
Firmicutes–Erysipelotrichia–Erysipelotrichales–Erysipelotrichaceae–Allobaculum
Firmicutes–Erysipelotrichia–Erysipelotrichales–Erysipelotrichaceae–Catenibacterium
Firmicutes–Erysipelotrichia–Erysipelotrichales–Erysipelotrichaceae–Clostridium XVIII
Firmicutes–Erysipelotrichia–Erysipelotrichales–Erysipelotrichaceae–Coprobacillus
Firmicutes–Erysipelotrichia–Erysipelotrichales–Erysipelotrichaceae–Erysipelotrichaceae_incertae_sedis
Firmicutes–Erysipelotrichia–Erysipelotrichales–Erysipelotrichaceae–Holdemania
Firmicutes–Erysipelotrichia–Erysipelotrichales–Erysipelotrichaceae–Solobacterium
Firmicutes–Erysipelotrichia–Erysipelotrichales–Erysipelotrichaceae–Turicibacter
Firmicutes–Erysipelotrichia–Erysipelotrichales–Erysipelotrichaceae–ud–Erysipelotrichaceae
Firmicutes–Negativicutes–Selenomonadales–Acidaminococcaceae–Acidaminococcus
Firmicutes–Negativicutes–Selenomonadales–Acidaminococcaceae–Phascolarctobacterium
Firmicutes–Negativicutes–Selenomonadales–Acidaminococcaceae–ud–Acidaminococcaceae
Firmicutes–Negativicutes–Selenomonadales–ud–Selenomonadales–ud–Selenomonadales
Firmicutes–Negativicutes–Selenomonadales–Veillonellaceae–Allisonella
Firmicutes–Negativicutes–Selenomonadales–Veillonellaceae–Dialister
Firmicutes–Negativicutes–Selenomonadales–Veillonellaceae–Megasphaera
Firmicutes–Negativicutes–Selenomonadales–Veillonellaceae–Mitsuokella
Firmicutes–Negativicutes–Selenomonadales–Veillonellaceae–Selenomonas
Firmicutes–Negativicutes–Selenomonadales–Veillonellaceae–ud–Veillonellaceae
Firmicutes–Negativicutes–Selenomonadales–Veillonellaceae–Veillonella
Firmicutes–ud–Firmicutes–ud–Firmicutes–ud–Firmicutes
Phylum–Class–Order–Family–Genus

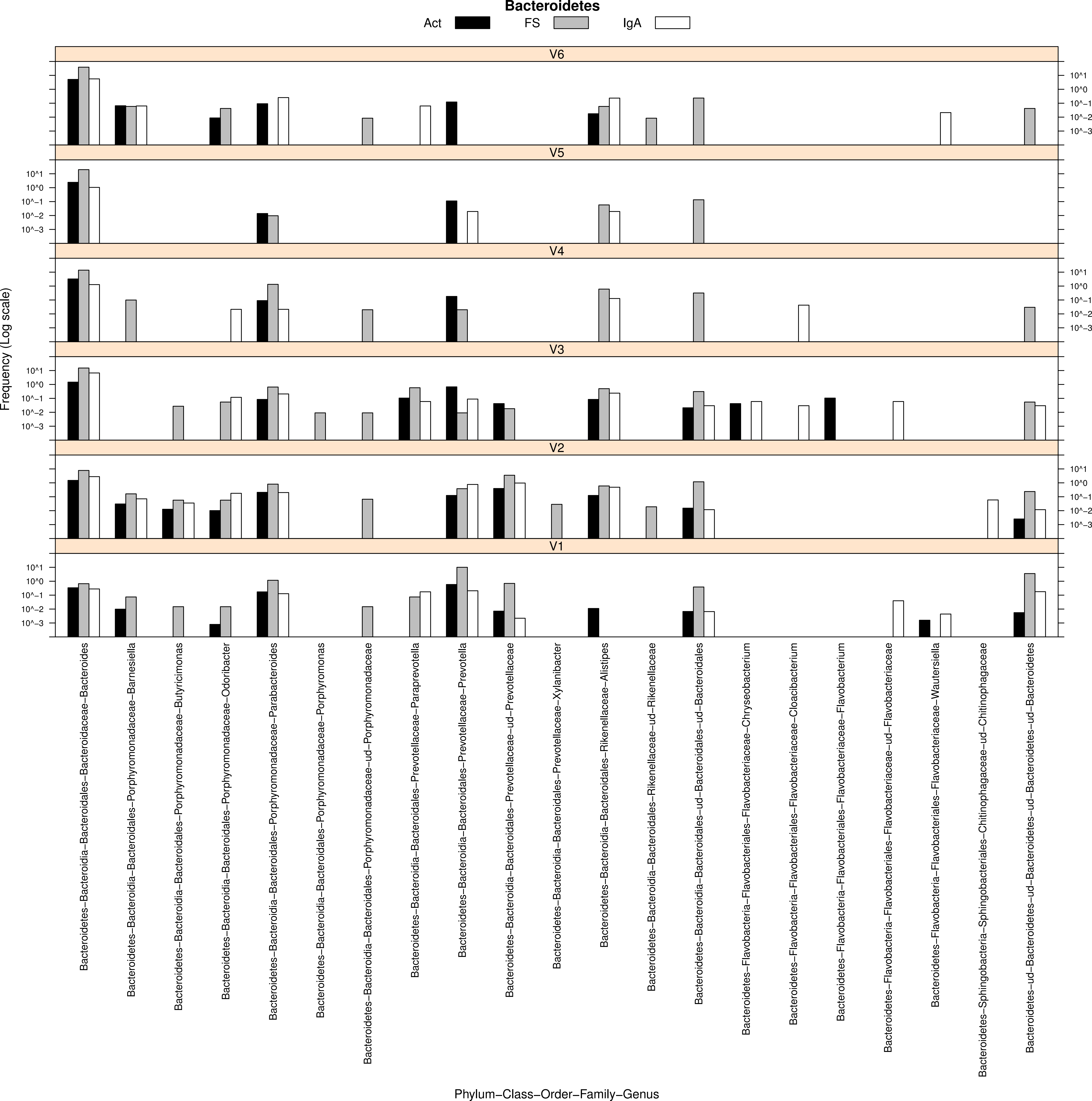
Bacteroidetes
Act
FS
IgA
V6
V5
V4
V3
V2
V1
10^1
10^0
10^−1
10^−2
10^−3
Frequency (Log scale)
Bacteroidetes−Bacteroidia−Bacteroidales−Bacteroidaceae−Bacteroides
Bacteroidetes−Bacteroidia−Bacteroidales−Porphyromonadaceae−Barnesiella
Bacteroidetes−Bacteroidia−Bacteroidales−Porphyromonadaceae−Butyricimonas
Bacteroidetes−Bacteroidia−Bacteroidales−Porphyromonadaceae−Odoribacter
Bacteroidetes−Bacteroidia−Bacteroidales−Porphyromonadaceae−Parabacteroides
Bacteroidetes−Bacteroidia−Bacteroidales−Porphyromonadaceae−Porphyromonas
Bacteroidetes−Bacteroidia−Bacteroidales−Porphyromonadaceae−ud−Porphyromonadaceae
Bacteroidetes−Bacteroidia−Bacteroidales−Prevotellaceae−Paraprevotella
Bacteroidetes−Bacteroidia−Bacteroidales−Prevotellaceae−Prevotella
Bacteroidetes−Bacteroidia−Bacteroidales−Prevotellaceae−ud−Prevotellaceae
Bacteroidetes−Bacteroidia−Bacteroidales−Prevotellaceae−Xylanibacter
Bacteroidetes−Bacteroidia−Bacteroidales−Rikenellaceae−Alistipes
Bacteroidetes−Bacteroidia−Bacteroidales−Rikenellaceae−ud−Rikenellaceae
Bacteroidetes−Bacteroidia−Bacteroidales−ud−Bacteroidales
Bacteroidetes−Flavobacteria−Flavobacteriales−Flavobacteriaceae−Chryseobacterium
Bacteroidetes−Flavobacteria−Flavobacteriales−Flavobacteriaceae−Cloacibacterium
Bacteroidetes−Flavobacteria−Flavobacteriales−Flavobacteriaceae−Flavobacterium
Bacteroidetes−Flavobacteria−Flavobacteriales−Flavobacteriaceae−ud−Flavobacteriaceae
Bacteroidetes−Flavobacteria−Flavobacteriales−Flavobacteriaceae−Wautersiella
Bacteroidetes−Sphingobacteria−Sphingobacteriales−Chitinophagaceae−ud−Chitinophagaceae
Bacteroidetes−ud−Bacteroidetes−ud−Bacteroidetes−ud−Bacteroidetes
Phylum−Class−Order−Family−Genus

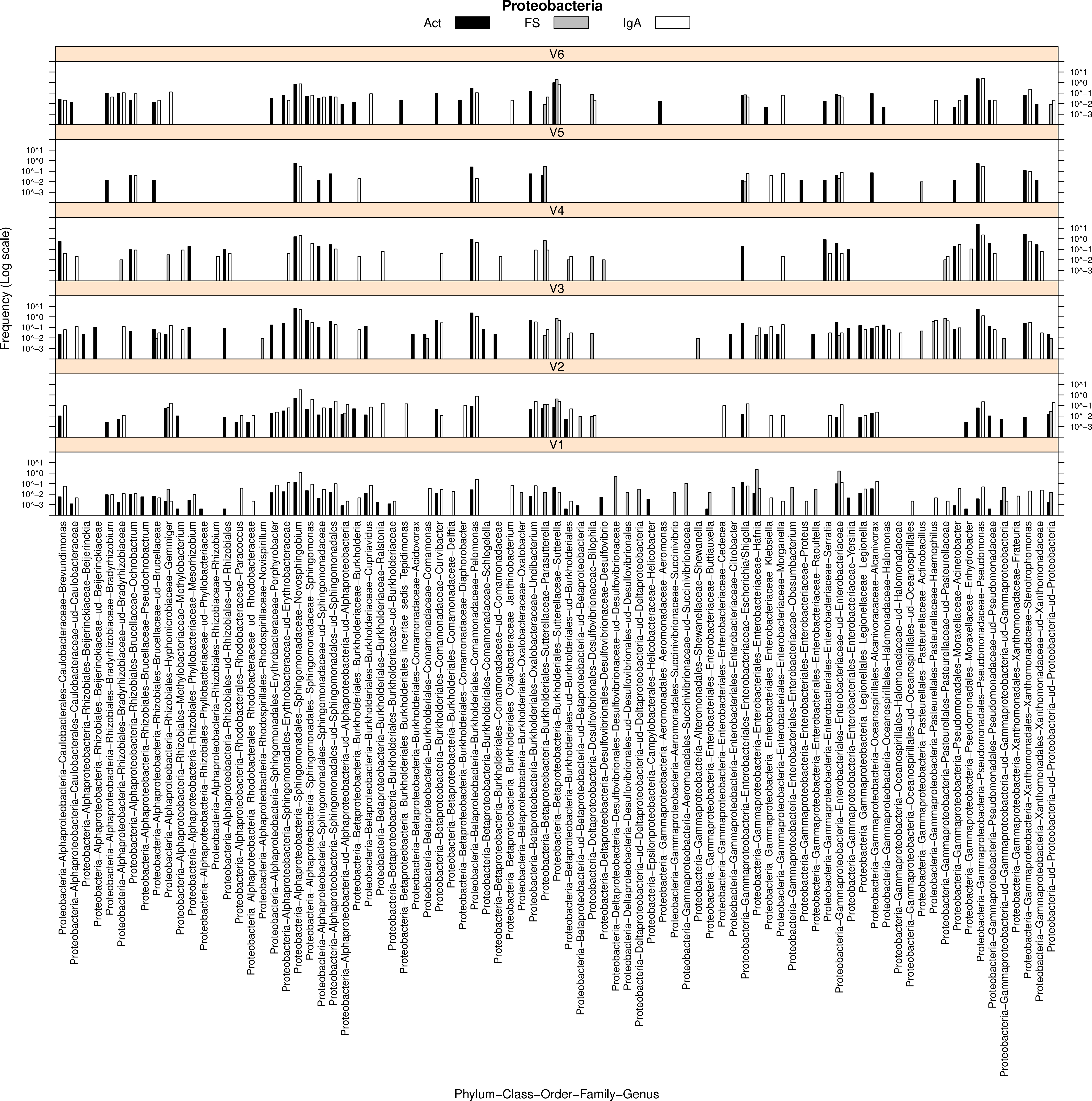
Proteobacteria
Act
FS
IgA
V6
V5
V4
V3
V2
V1
10^1
10^0
10^−1
10^−2
10^−3
Frequency (Log scale)
Proteobacteria–Alphaproteobacteria–Caulobacterales–Caulobacteraceae–Brevundimonas
Proteobacteria–Alphaproteobacteria–Caulobacterales–Caulobacteraceae–ud–Caulobacteraceae
Proteobacteria–Alphaproteobacteria–Rhizobiales–Beijerinckiaceae–Beijerinckia
Proteobacteria–Alphaproteobacteria–Rhizobiales–Beijerinckiaceae–ud–Beijerinckiaceae
Proteobacteria–Alphaproteobacteria–Rhizobiales–Bradyrhizobiaceae–Bradyrhizobium
Proteobacteria–Alphaproteobacteria–Rhizobiales–Bradyrhizobiaceae–ud–Bradyrhizobiaceae
Proteobacteria–Alphaproteobacteria–Rhizobiales–Brucellaceae–Ochrobactrum
Proteobacteria–Alphaproteobacteria–Rhizobiales–Brucellaceae–Pseudochrobactrum
Proteobacteria–Alphaproteobacteria–Rhizobiales–Brucellaceae–ud–Brucellaceae
Proteobacteria–Alphaproteobacteria–Rhizobiales–Hyphomicrobiaceae–Gemmiger
Proteobacteria–Alphaproteobacteria–Rhizobiales–Methylobacteriaceae–Methylobacterium
Proteobacteria–Alphaproteobacteria–Rhizobiales–Phyllobacteriaceae–Mesorhizobium
Proteobacteria–Alphaproteobacteria–Rhizobiales–Phyllobacteriaceae–ud–Phyllobacteriaceae
Proteobacteria–Alphaproteobacteria–Rhizobiales–Rhizobiaceae–Rhizobium
Proteobacteria–Alphaproteobacteria–Rhizobiales–ud–Rhizobiales–ud–Rhizobiales
Proteobacteria–Alphaproteobacteria–Rhodobacterales–Rhodobacteraceae–Paracoccus
Proteobacteria–Alphaproteobacteria–Rhodobacterales–Rhodobacteraceae–ud–Rhodobacteraceae
Proteobacteria–Alphaproteobacteria–Rhodospirillales–Rhodospirillaceae–Novispirillum
Proteobacteria–Alphaproteobacteria–Sphingomonadales–Erythrobacteraceae–Porphyrobacter
Proteobacteria–Alphaproteobacteria–Sphingomonadales–Erythrobacteraceae–ud–Erythrobacteraceae
Proteobacteria–Alphaproteobacteria–Sphingomonadales–Sphingomonadaceae–Novosphingobium
Proteobacteria–Alphaproteobacteria–Sphingomonadales–Sphingomonadaceae–Sphingomonas
Proteobacteria–Alphaproteobacteria–Sphingomonadales–Sphingomonadaceae–ud–Sphingomonadaceae
Proteobacteria–Alphaproteobacteria–Sphingomonadales–ud–Sphingomonadales–ud–Sphingomonadales
Proteobacteria–Alphaproteobacteria–ud–Alphaproteobacteria–ud–Alphaproteobacteria
Proteobacteria–Betaproteobacteria–Burkholderiales–Burkholderiaceae–Burkholderia
Proteobacteria–Betaproteobacteria–Burkholderiales–Burkholderiaceae–Cupriavidus
Proteobacteria–Betaproteobacteria–Burkholderiales–Burkholderiaceae–Ralstonia
Proteobacteria–Betaproteobacteria–Burkholderiales–Burkholderiaceae–ud–Burkholderiaceae
Proteobacteria–Betaproteobacteria–Burkholderiales–Burkholderiales_incertae_sedis–Tepidimonas
Proteobacteria–Betaproteobacteria–Burkholderiales–Comamonadaceae–Acidovorax
Proteobacteria–Betaproteobacteria–Burkholderiales–Comamonadaceae–Comamonas
Proteobacteria–Betaproteobacteria–Burkholderiales–Comamonadaceae–Curvibacter
Proteobacteria–Betaproteobacteria–Burkholderiales–Comamonadaceae–Delftia
Proteobacteria–Betaproteobacteria–Burkholderiales–Comamonadaceae–Diaphorobacter
Proteobacteria–Betaproteobacteria–Burkholderiales–Comamonadaceae–Pelomonas
Proteobacteria–Betaproteobacteria–Burkholderiales–Comamonadaceae–Schlegelella
Proteobacteria–Betaproteobacteria–Burkholderiales–Comamonadaceae–ud–Comamonadaceae
Proteobacteria–Betaproteobacteria–Burkholderiales–Oxalobacteraceae–Janthinobacterium
Proteobacteria–Betaproteobacteria–Burkholderiales–Oxalobacteraceae–Oxalobacter
Proteobacteria–Betaproteobacteria–Burkholderiales–Oxalobacteraceae–Undibacterium
Proteobacteria–Betaproteobacteria–Burkholderiales–Sutterellaceae–Parasutterella
Proteobacteria–Betaproteobacteria–Burkholderiales–Sutterellaceae–Sutterella
Proteobacteria–Betaproteobacteria–Burkholderiales–ud–Burkholderiales–ud–Burkholderiales
Proteobacteria–Betaproteobacteria–ud–Betaproteobacteria–ud–Betaproteobacteria
Proteobacteria–Deltaproteobacteria–Desulfovibrionales–Desulfovibrionaceae–Bilophila
Proteobacteria–Deltaproteobacteria–Desulfovibrionales–Desulfovibrionaceae–Desulfovibrio
Proteobacteria–Deltaproteobacteria–Desulfovibrionales–Desulfovibrionaceae–ud–Desulfovibrionaceae
Proteobacteria–Deltaproteobacteria–Desulfovibrionales–ud–Desulfovibrionales–ud–Desulfovibrionales
Proteobacteria–Deltaproteobacteria–ud–Deltaproteobacteria–ud–Deltaproteobacteria
Proteobacteria–Epsilonproteobacteria–Campylobacterales–Helicobacteraceae–Helicobacter
Proteobacteria–Gammaproteobacteria–Aeromonadales–Aeromonadaceae–Aeromonas
Proteobacteria–Gammaproteobacteria–Aeromonadales–Succinivibrionaceae–Succinivibrio
Proteobacteria–Gammaproteobacteria–Aeromonadales–Succinivibrionaceae–ud–Succinivibrionaceae
Proteobacteria–Gammaproteobacteria–Alteromonadales–Shewanellaceae–Shewanella
Proteobacteria–Gammaproteobacteria–Enterobacteriales–Enterobacteriaceae–Buttiauxella
Proteobacteria–Gammaproteobacteria–Enterobacteriales–Enterobacteriaceae–Cedecea
Proteobacteria–Gammaproteobacteria–Enterobacteriales–Enterobacteriaceae–Citrobacter
Proteobacteria–Gammaproteobacteria–Enterobacteriales–Enterobacteriaceae–Escherichia/Shigella
Proteobacteria–Gammaproteobacteria–Enterobacteriales–Enterobacteriaceae–Hafnia
Proteobacteria–Gammaproteobacteria–Enterobacteriales–Enterobacteriaceae–Klebsiella
Proteobacteria–Gammaproteobacteria–Enterobacteriales–Enterobacteriaceae–Morganella
Proteobacteria–Gammaproteobacteria–Enterobacteriales–Enterobacteriaceae–Obesumbacterium
Proteobacteria–Gammaproteobacteria–Enterobacteriales–Enterobacteriaceae–Proteus
Proteobacteria–Gammaproteobacteria–Enterobacteriales–Enterobacteriaceae–Raoultella
Proteobacteria–Gammaproteobacteria–Enterobacteriales–Enterobacteriaceae–Serratia
Proteobacteria–Gammaproteobacteria–Enterobacteriales–Enterobacteriaceae–ud–Enterobacteriaceae
Proteobacteria–Gammaproteobacteria–Enterobacteriales–Enterobacteriaceae–Yersinia
Proteobacteria–Gammaproteobacteria–Legionellales–Legionellaceae–Legionella
Proteobacteria–Gammaproteobacteria–Oceanospirillales–Alcanivoracaceae–Alcanivorax
Proteobacteria–Gammaproteobacteria–Oceanospirillales–Halomonadaceae–Halomonas
Proteobacteria–Gammaproteobacteria–Oceanospirillales–Halomonadaceae–ud–Halomonadaceae
Proteobacteria–Gammaproteobacteria–Oceanospirillales–ud–Oceanospirillales–ud–Oceanospirillales
Proteobacteria–Gammaproteobacteria–Pasteurellales–Pasteurellaceae–Actinobacillus
Proteobacteria–Gammaproteobacteria–Pasteurellales–Pasteurellaceae–Haemophilus
Proteobacteria–Gammaproteobacteria–Pasteurellales–Pasteurellaceae–ud–Pasteurellaceae
Proteobacteria–Gammaproteobacteria–Pseudomonadales–Moraxellaceae–Acinetobacter
Proteobacteria–Gammaproteobacteria–Pseudomonadales–Moraxellaceae–Enhydrobacter
Proteobacteria–Gammaproteobacteria–Pseudomonadales–Pseudomonadaceae–Pseudomonas
Proteobacteria–Gammaproteobacteria–Pseudomonadales–Pseudomonadaceae–ud–Pseudomonadaceae
Proteobacteria–Gammaproteobacteria–ud–Gammaproteobacteria–ud–Gammaproteobacteria
Proteobacteria–Gammaproteobacteria–Xanthomonadales–Xanthomonadaceae–Frateuria
Proteobacteria–Gammaproteobacteria–Xanthomonadales–Xanthomonadaceae–Stenotrophomonas
Proteobacteria–Gammaproteobacteria–Xanthomonadales–Xanthomonadaceae–ud–Xanthomonadaceae
Proteobacteria–ud–Proteobacteria–ud–Proteobacteria
Phylum–Class–Order–Family–Genus

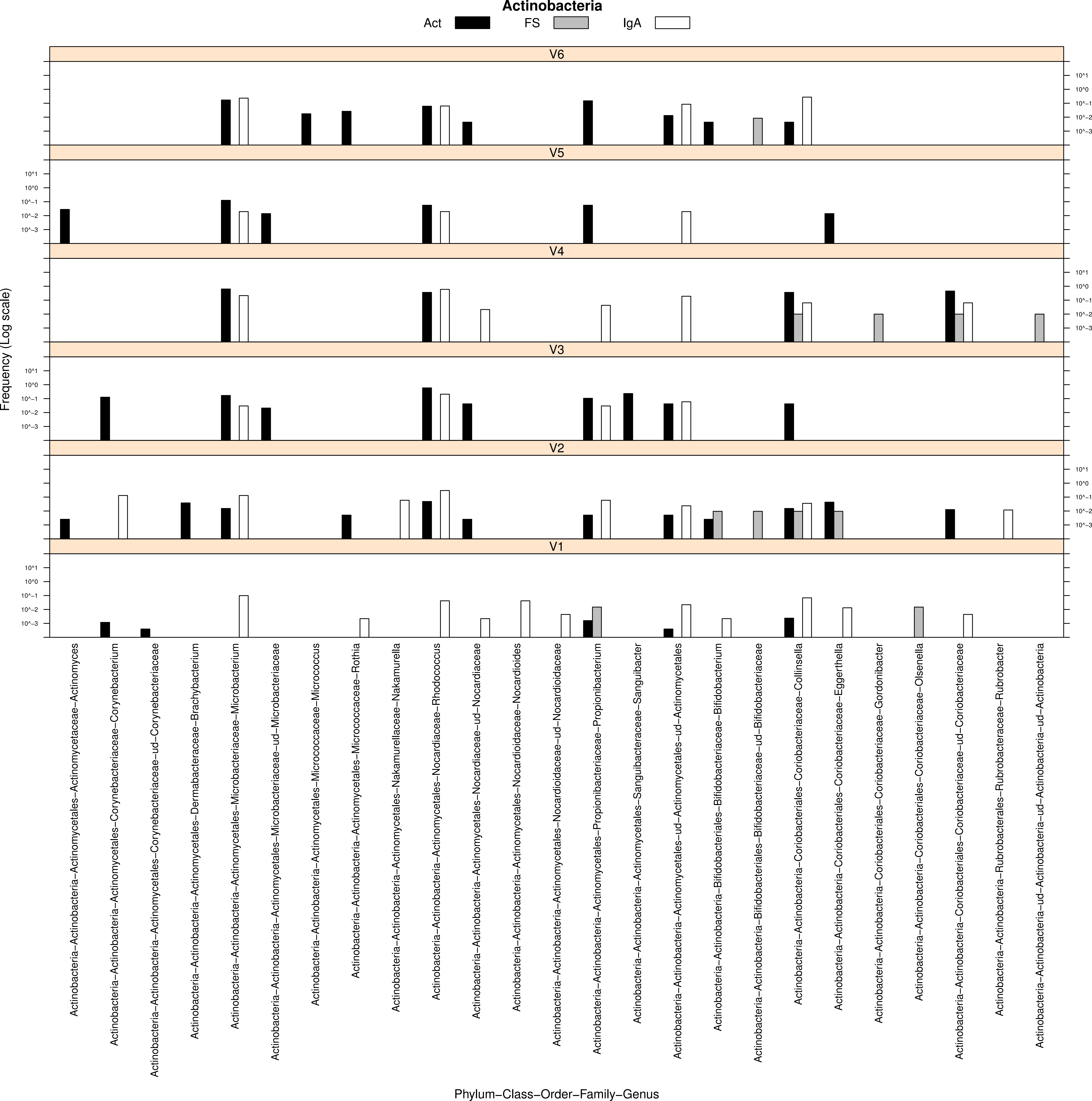

Actinobacteria
Act
FS
IgA
V6
V5
V4
V3
V2
V1
10^1
10^0
10^−1
10^−2
10^−3
Frequency (Log scale)
Actinobacteria–Actinobacteria–Actinomycetales–Actinomycetaceae–Actinomyces
Actinobacteria–Actinobacteria–Actinomycetales–Corynebacteriaceae–Corynebacterium
Actinobacteria–Actinobacteria–Actinomycetales–Corynebacteriaceae–ud–Corynebacteriaceae
Actinobacteria–Actinobacteria–Actinomycetales–Dermabacteraceae–Brachybacterium
Actinobacteria–Actinobacteria–Actinomycetales–Microbacteriaceae–Microbacterium
Actinobacteria–Actinobacteria–Actinomycetales–Microbacteriaceae–ud–Microbacteriaceae
Actinobacteria–Actinobacteria–Actinomycetales–Micrococcaceae–Micrococcus
Actinobacteria–Actinobacteria–Actinomycetales–Micrococcaceae–Rothia
Actinobacteria–Actinobacteria–Actinomycetales–Nakamurellaceae–Nakamurella
Actinobacteria–Actinobacteria–Actinomycetales–Nocardiaceae–Rhodococcus
Actinobacteria–Actinobacteria–Actinomycetales–Nocardiaceae–ud–Nocardiaceae
Actinobacteria–Actinobacteria–Actinomycetales–Nocardioidaceae–Nocardioides
Actinobacteria–Actinobacteria–Actinomycetales–Nocardioidaceae–ud–Nocardioidaceae
Actinobacteria–Actinobacteria–Actinomycetales–Propionibacteriaceae–Propionibacterium
Actinobacteria–Actinobacteria–Actinomycetales–Sanguibacteraceae–Sanguibacter
Actinobacteria–Actinobacteria–Actinomycetales–ud–Actinomycetales
Actinobacteria–Actinobacteria–Bifidobacteriales–Bifidobacteriaceae–Bifidobacterium
Actinobacteria–Actinobacteria–Bifidobacteriales–Bifidobacteriaceae–ud–Bifidobacteriaceae
Actinobacteria–Actinobacteria–Coriobacteriales–Coriobacteriaceae–Collinsella
Actinobacteria–Actinobacteria–Coriobacteriales–Coriobacteriaceae–Eggerthella
Actinobacteria–Actinobacteria–Coriobacteriales–Coriobacteriaceae–Gordonibacter
Actinobacteria–Actinobacteria–Coriobacteriales–Coriobacteriaceae–Olsenella
Actinobacteria–Actinobacteria–Coriobacteriales–Coriobacteriaceae–ud–Coriobacteriaceae
Actinobacteria–Actinobacteria–Rubrobacterales–Rubrobacteraceae–Rubrobacter
Actinobacteria–Actinobacteria–ud–Actinobacteria–ud–Actinobacteria
Phylum–Class–Order–Family–Genus

Figure 3: Control flow cytometry bi-plots. Top panel shows size (X axis) versus complexity (Y axis) of unstained cells compared with 3, 6, and 10 micrometer beads. In bottom panel X axis shows fluorescence emitted by DNA labelled with SYTO62 dye. Y axis represents the fluorescence emitted by anti-S-IgA immunoglobulin labelled with FITC. Negative control panel reports unstained cells. Isotype control reports cell stained with SYTO62 (DNA) and mouse-anti-IgA as isotype control in order to identify unspecific hybridisation region. Experiment plot shows the human-anti-S-IgA hybridised cells. Green area indicates the unspecific region obtained by isotype control.

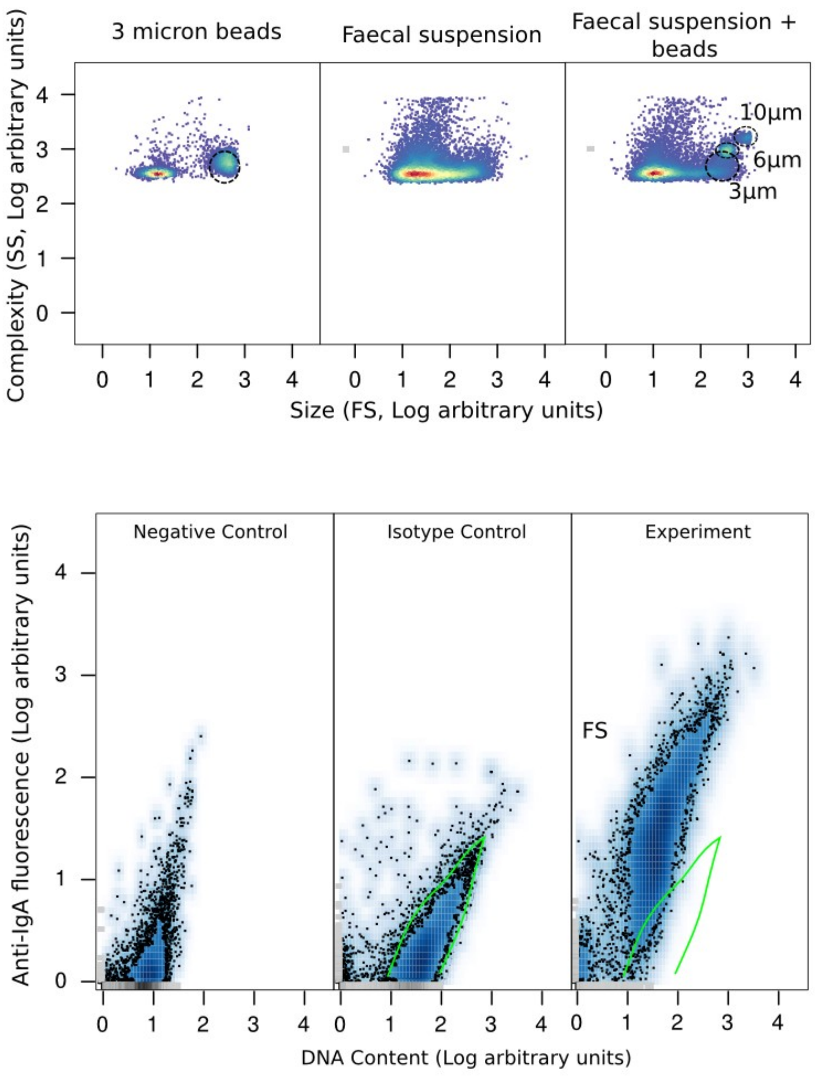

Figure 4: Flow Cytometry bi-plot. X axis show fluorescence emitted by DNA labelled with Syto62 dye reported in arbitrary logarithmic units. First two columns show respectively control experiments without staining (Negative), and only DNA staining (Syto62) visible as an increment on the X axis. Third and fourth columns show the fluorescence emitted by anti-s-IgA staining against: mouse (isotype control) and human (experiment for sorting) respectively. S-IgA is shown on the Y axis while DNA staining is represented on the X axis. The fifth column reports pyronin-Y staining (Y axis) as a measure of cell activity. Every line corresponds to a volunteer sample. Three of the experimental files have been lost due to technical IT problems (NAs).

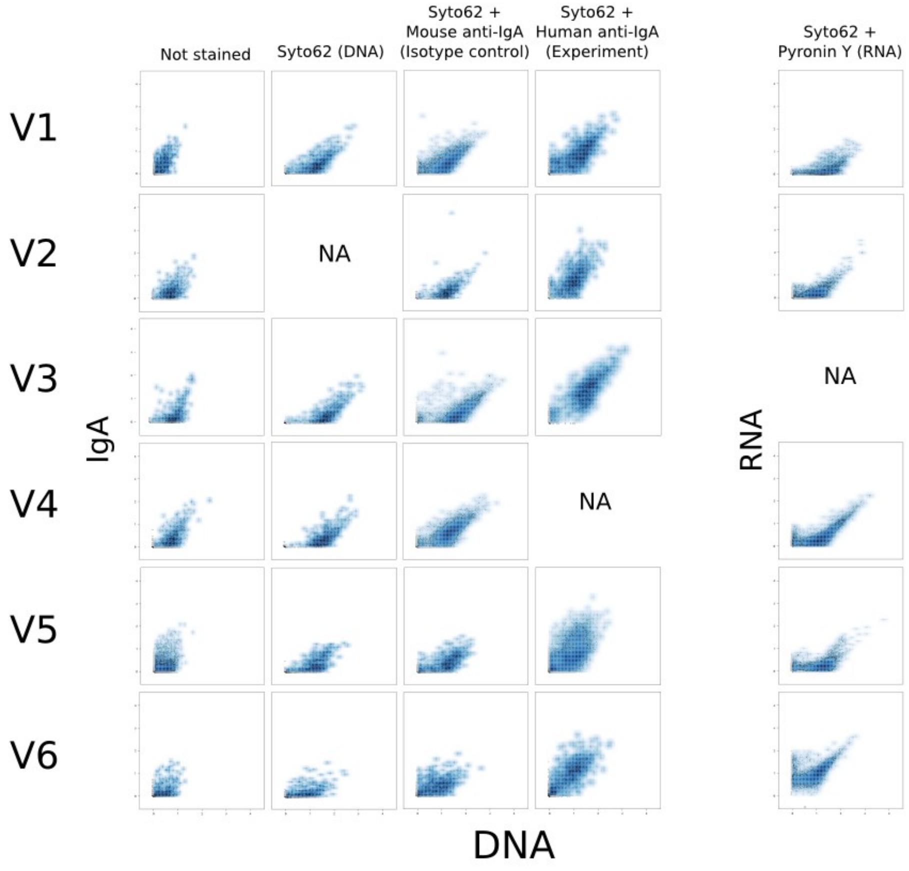

Supplement: Supplementary Information — Supplemental Information [file srep03515-s1.pdf]
